# Therapeutic targeting of inflammation in hypertension: from novel mechanisms to translational perspective

**DOI:** 10.1093/cvr/cvab330

**Published:** 2021-10-26

**Authors:** Eleanor C Murray, Ryszard Nosalski, Neil MacRitchie, Maciej Tomaszewski, Pasquale Maffia, David G Harrison, Tomasz J Guzik

**Affiliations:** Institute of Cardiovascular and Medical Sciences, College of Medical, Veterinary and Life Sciences, University of Glasgow, G12 8TA Glasgow, UK; Institute of Cardiovascular and Medical Sciences, College of Medical, Veterinary and Life Sciences, University of Glasgow, G12 8TA Glasgow, UK; Department of Internal Medicine, Collegium Medicum, Jagiellonian University, 31-008 Kraków, Poland; Centre for Immunobiology, Institute of Infection, Immunity and Inflammation, College of Medical, Veterinary and Life Sciences, University of Glasgow, G12 8TA Glasgow, UK; Division of Cardiovascular Sciences, Faculty of Medicine, Biology and Health, University of Manchester, M13 9PL Manchester, UK; Manchester Heart Centre and Manchester Academic Health Science Centre, Manchester University NHS Foundation Trust, M13 9WL Manchester, UK; Institute of Cardiovascular and Medical Sciences, College of Medical, Veterinary and Life Sciences, University of Glasgow, G12 8TA Glasgow, UK; Centre for Immunobiology, Institute of Infection, Immunity and Inflammation, College of Medical, Veterinary and Life Sciences, University of Glasgow, G12 8TA Glasgow, UK; Department of Pharmacy, University of Naples Federico II, 80131 Naples, Italy; Division of Clinical Pharmacology, Department of Medicine, Vanderbildt University Medical Centre, Nashville, 37232 TN, USA; Institute of Cardiovascular and Medical Sciences, College of Medical, Veterinary and Life Sciences, University of Glasgow, G12 8TA Glasgow, UK; Department of Internal Medicine, Collegium Medicum, Jagiellonian University, 31-008 Kraków, Poland

**Keywords:** Hypertension, Inflammation, Immune system, Immunomodulatory, Blood pressure

## Abstract

Both animal models and human observational and genetic studies have shown that immune and inflammatory mechanisms play a key role in hypertension and its complications. We review the effects of immunomodulatory interventions on blood pressure, target organ damage, and cardiovascular risk in humans. In experimental and small clinical studies, both non-specific immunomodulatory approaches, such as mycophenolate mofetil and methotrexate, and medications targeting T and B lymphocytes, such as tacrolimus, cyclosporine, everolimus, and rituximab, lower blood pressure and reduce organ damage. Mechanistically targeted immune interventions include isolevuglandin scavengers to prevent neo-antigen formation, co-stimulation blockade (abatacept, belatacept), and anti-cytokine therapies (e.g. secukinumab, tocilizumab, canakinumab, TNF-α inhibitors). In many studies, trial designs have been complicated by a lack of blood pressure-related endpoints, inclusion of largely normotensive study populations, polypharmacy, and established comorbidities. Among a wide range of interventions reviewed, TNF-α inhibitors have provided the most robust evidence of blood pressure lowering. Treatment of periodontitis also appears to deliver non-pharmacological anti-hypertensive effects. Evidence of immunomodulatory drugs influencing hypertension-mediated organ damage are also discussed. The reviewed animal models, observational studies, and trial data in humans, support the therapeutic potential of immune-targeted therapies in blood pressure lowering and in hypertension-mediated organ damage. Targeted studies are now needed to address their effects on blood pressure in hypertensive individuals.

## Graphical Abstract

**Figure:**
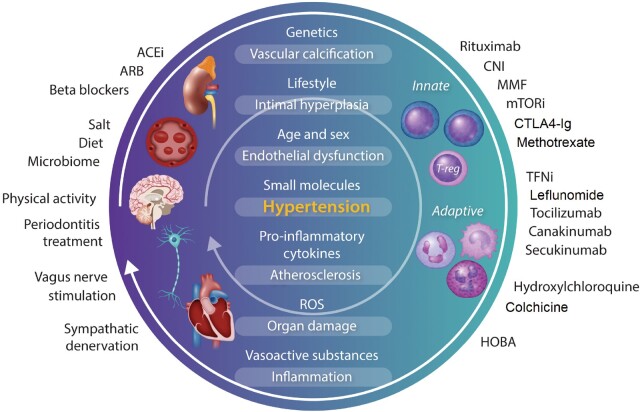

## 1. Introduction

In atherosclerosis, the role of inflammation is well defined,^1–5^ and a co-existing chronic inflammatory condition such as rheumatoid arthritis (RA), inflammatory bowel disease, ankylosing spondylitis, or psoriasis is considered an additional risk factor, including in ESC Cardiovascular Disease Prevention guidelines.^6–8^ Anti-inflammatory therapies are recommended in such patients,^6^ and targeting inflammation to improve cardiovascular outcomes has been supported by recent clinical trials such as CANTOS, COLCOT, and LoDoCo2.^9–12^ Hypertension is the most common cardiovascular risk factor worldwide.^13^ For more than half a century, immune cells have been observed to infiltrate the kidney and vasculature of hypertensive humans and animals with experimental hypertension, and increasing evidence indicates that immune and inflammatory mechanisms promote this disease. It is therefore essential to identify the clinically permissible therapeutic interventions that address inflammatory targets in hypertension, and patient populations that would benefit from such treatment. While basic and translational evidence suggests that interfering in immune-inflammatory processes may aid in control of blood pressure (BP) and prevention of target organ damage,^14–17^ the clinical evidence for these interventions has not been systematically analysed. Accordingly, we review potential immune therapeutic targets to identify approaches for which well-designed clinical studies may prove fruitful.

## 2. Immune and inflammatory targets for treatment of hypertension

**Figure 1:**
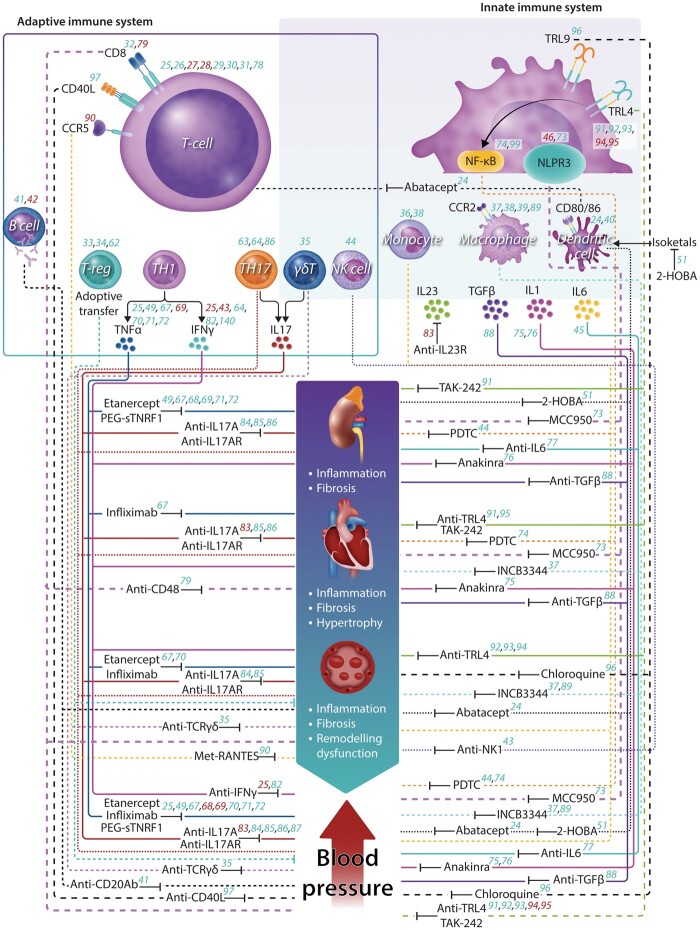
Role of the immune system in the pathogenesis of experimental hypertension and potential immunomodulators for the treatment of hypertension and cardiovascular organ damage. Animal studies implicate virtually all immune cell subsets (dash lines) and cytokines (solid lines) in the pathogenesis of hypertension and target organ damage. Initially, classical immunosuppressants such as mycophenolate^59^^,^^60^ or rapamycin^58^ showed improvement in renal damage and blood pressure regulation, by non-specific mechanisms. The introduction of cell/cytokine-specific immunomodulators (small-molecule inhibitors, antibodies, antagonists or scavengers) with beneficial effect in hypertension and hypertension-mediated organ damage, emphasize the potential use of immunomodulators as a pharmacological tool. More details about the inhibitors are presented in Table1. Numbers indicate references represent a positive (green) or negative (red) effect. Legend: CD, cluster of differentiation; CCR, chemokine receptor; Treg, T-regulatory cell; TH, T-helper cell; IL, interleukin; TNF-α, tumour necrosis factor alfa; NF-κB, Nuclear factor kappa B; IFN-γ, interferon γ; NLPR3, NOD-like receptor family, pyrin domain-containing protein 3; TGF-β, transforming growth factor beta; TLR, Toll-like receptor; PEG-sTNFR1, PEGylated soluble tumour necrosis factor receptor 1; TAK-242, inhibitor of TLR4 signalling; 2-HOBA, 2-hydroxybenzylamine; MCC950, small-molecule inhibitor of the NLRP3 pathway; INCB3344, CCR2 antagonist; Met-RANTES, CCR5 antagonist.

Inflammation and immune activation were first implicated as being involved in hypertension through the work of Grollman, Okuda, Svendsen,^18–21^ and Olsen.^22^^,^^23^ In the last decade, new research has begun to reveal the mechanisms that explain this.^17^ Using animal models of genetic and pharmacological targeting, the regulatory role of T cells,^24–34^ γδ cells,^35^ monocytes/macrophages,^36–39^ dendritic cells (DC),^40^ B cells,^41^^,^^42^ NK cells,^43^ as well as other components of a complex immuno-inflammatory network have been assessed.^17^^,^^44–48^ The initiation of inflammation in hypertension appears to be associated with oxidative stress and redox-dependent mechanisms within the vascular and renal tissues.^49^^,^^50^ These lead to generation of neo-antigens,^51^ damage-associated molecular patterns,^52^ and neuroimmune mechanisms^53^^,^^54^ that trigger maladaptive immune responses, which compound hypertension and its' associated organ damage. Although antigen(s) responsible for activation of adaptive immunity have not been definitively identified, potential candidates are isolevuglandin (isoLG) adducted proteins. IsoLGs are oxidation products of arachidonic acid that rapidly ligate lysines on self-proteins and accumulate in antigen-presenting cells and are presented within major histocompatibility complexes. These activate a subset of CD4^+^ and CD8^+^ T cells. Importantly, the selective isoLG scavenger 2-hydroxybenzylamine can prevent immune activation and lower BP in several animal models of hypertension.^51^ Data from both animal and human studies also identify HSP70 as a potential auto-antigen.^55^ Numerous animal studies in a wide range of models, reviewed previously,^56–58^ highlight that immunomodulating inflammatory activation and effector cytokine release may curb BP increases and lessen development of vascular, cardiac, and renal damage.^59–66^ Many of these studies have employed germline knockout animals. In addition, small molecule or neutralizing antibodies that target immune mediators have been used to determine the effect of selective blockade on experimental hypertension (*Table 1*). These have targeted both the innate (e.g. IL-1, TLR4) and adaptive (e.g. IL-17, CD80/86) immune system. Careful analysis of these studies helps identify potential therapeutic targets, but also highlights the impact of treatment protocol and animal model selection for BP and target organ damage outcomes (*Table 1* and *Figure 1*).

**Table 1:** Key findings relevant to the relationship between the immune system and hypertension arising from animal models

Immune target and therapeutic agent	Model	Result relative to non-treated mice	References
**TNF-α** Infliximab (anti-TNF-α neutralizing Ab)	SHR rat	↓ BP ↓ Cardiac hypertrophy ↓ Vascular inflammation	Filho *et al*.^67^
Etanercept (TNF-inhibitor)	Rat (8% NaCl diet + 14 days ang-II)	↓ Renal inflammation/damage Slowed but did not prevent rise in BP	Elmarakby *et al*.^68^
Etanercept	Mice infused with ang-II for 14 days	↓ BP	Guzik *et al*.^25^
Etanercept	Spontaneously hypertensive dTGR rats	↓ Renal inflammation/damage ↓ Mortality ↔ BP	Muller *et al*.^69^
Etanercept	Dahl salt-sensitive rat with renal interstitial administration of etanercept	↓ BP ↓ renal damage	Huang *et al*.^49^
Etanercept	High fructose-fed rats	↓ BP ↓ Endothelial dysfunction	Tran *et al*.^70^
Etanercept	Mouse model of SLE	↓ BP ↓ Renal inflammation/damage	Venegas-Pont *et al.*^71^
PEG-sTNFR1 (TNF inhibitor)	Renal mass reduction induced renal failure in rats	↓ BP ↓ Renal inflammation/damage	Therrien *et al*.^72^
**NLRP3** MCC950 (NLRP3 inhibitor)	Uni-nephrectomized wild-type mice treated with DOCA-salt up to 28 days	↓ BP ↓ Cardiac hypertrophy ↓ Renal inflammation/damage	Krishnan *et al*.^73^
**NF-κb** PDTC	SHR rats	↓ BP ↓ Renal inflammation	Rodríguez-Iturbe *et al*.^44^
PDTC	2K1C rats	↓ BP ↓ Cardiac hypertrophy/fibrosis	Cau *et al*.^74^
**IL-1R** Anakinra (IL-1R antagonist)	Mice treated with ang-II for 21 days	↓ BP ↓ Cardiac hypertrophy	Zhang *et al.*^75^
Anakinra	Uni-nephrectomized mice treated with DOCA-salt in drinking water for 21 days	↓ BP ↓ Renal fibrosis	Ling *et al*.^76^
**IL-6** Neutralizing anti-IL-6 Ab	Dahl salt-sensitive rats fed 4% NaCl for up to 11 days	↓ BP ↓ Renal inflammation/damage	Hashmat *et al*.^77^
**T cells** Anti-CD3 Ab	Mouse model of SLE	↓ BP ↓ Autoantibodies ↓ BP	Mathis *et al*.^78^
**CD8 T cells** Anti-CD8 Ab	Mice treated with ang-II for 14 days	↔ BP ↓ Cardiac inflammation and fibrosis	Ma *et al*.^79^
**γδ T cells** Anti-γδ T cell Ab	Mice treated with ang-II for 7 or 14 days	↓ BP ↓ Endothelial dysfunction	Caillon *et al*.^35^
**Tregs** IL-2/Anti-IL-2 Ab complex	Mice treated with ang-II for 14 days	↔ BP ↓ Aortic remodelling ↓ Aortic stiffness	Majeed *et al*.^80^
IL-2/Anti-IL-2 Ab complex	Transverse aortic constriction (TAC) in mice	↔ BP ↓ Cardiac hypertrophy and dysfunction	Wang *et al*.^81^
**IFN- γ** Neutralizing anti-IFN-γ Ab	Wild-type mice treated with ang-II for 14 days	↔ BP	Guzik *et al*.^25^
Neutralizing anti-IFN-γ Ab	Mice with T-cell restricted overexpression of mineralocorticoid receptor (TMROV mice) treated with ang-II for 21 days	↓ BP	Sun *et al*.^82^
**IL-17** Neutralizing anti-IL-17A Ab	Wild-type mice treated with ang-II for 14 days	↔ BP and cardiac hypertrophy	Markó *et al*.^83^
Neutralizing anti-IL-17A Ab	Rats treated with anti-IL-17A Ab for 28 days using the DOCA-salt model	↓ BP ↓ Target organ damage	Amador *et al*.^84^
Neutralizing anti-IL-17A Ab	Calcineurin-inhibitor treated mice	↓ BP ↓ Endothelial dysfunction ↓ Renal damage	Chiasson *et al*.^85^
Neutralizing anti-IL-17A, IL-17F or Il-17RA	Wild-type mice treated for 28 days with ang-II (14 days with Ab treatment)	IL-17A/IL-17R: ↓ BP ↓ Renal inflammation/damage Il-17F: No significant change	Saleh *et al*.^86^
IL-17 soluble receptor C	Preeclampsia rat model	↓ BP ↓ Oxidative stress	Cornelius *et al*.^87^
**IL-23** Neutralizing anti-IL-21R Ab	Wild-type mice treated with ang-II for 14 days	↔ BP and cardiac hypertrophy	Marko *et al*.^83^
**TGF-β** Neutralizing anti-TGF-β Ab (1D11)	Dahl salt-sensitive rat fed 4% NaCl for 21-28 days	↓ BP ↓ Renal injury ↓ Cardiac damage	Murphy *et al*.^88^
**B cells** Anti-CD20 Ab	Wild-type mice treated with ang-II for 28 days	↓ BP	Chan *et al*.^41^
**CCR2** INCB3344 (CCR2 antagonist)	Wild-type mice were uni-nephrectomized and treated with DOCA-salt for 21 days	↓ BP ↓ Vascular inflammation	Chan *et al*.^89^
INCB3344	Wild-type mice treated with ang-II for 28 days (21 days with CCR2 antagonist)	↓ BP ↓ Vascular inflammation/fibrosis ↓ Cardiac hypertrophy	Moore *et al*.^37^
**CCR5** Met-RANTES (CCR5 antagonist)	Wild-type mice infused with ang-II for 14 days.	↓ Vascular inflammation and dysfunction ↔ BP	Mikolajczyk *et al*.^90^
**TLR-4** TAK-242 (TLR-4 antagonist)	Rats infused with Aldo-salt for 28 days	↓ BP ↓ Cardiac hypertrophy ↓ Renal damage	De Batista *et al*.^91^
Neutralizing anti‐TLR4 Ab	Wild-type mice treated with ang-II for 14 days	↓ BP ↓ Vascular inflammation and remodelling	Hernanz *et al*.^92^
Neutralizing anti‐TLR4 Ab	SHR rat	↓ BP ↓ Vascular reactivity	Bomfin *et al*.^93^
Neutralizing anti‐TLR4 Ab	Mice treated for 28 days with ang-II (14 days with Ab treatment)	↓ Vascular dysfunction ↔ BP	Nunes *et al*.^94^
Neutralizing anti‐TLR4 Ab	SHR rat	↔ BP ↓ Cardiac hypertrophy ↓ Cardiac inflammation	Echem *et al*.^95^
**TLR-9** Chloroquine (TLR-9 inhibitor + pleiotropic effects)	SHR rat	↓ BP ↓ Vascular and systemic inflammation	McCarthy *et al*.^96^
**CD80/CD86** CTLA4-Ig (Abatacept) (CD80/86 inhibitor)	Wild-type mice infused with ang-II for 14 days, or uni-nephrectomized and treated with DOCA-salt for 21 days	↓ BP ↓ Vascular and systemic inflammation	Vinh *et al*.^24^
**CD40L** Anti-CD40L Ab	Preeclampsia rat model	↓ BP ↓ Oxidative stress ↓ Endothelin-1 release	Cornelius *et al*.^97^
**Isoketals** 2-Hydroxybenzylamine (2-HOBA) (isoketal scavenger)	Wild-type mice were infused with ang-II for 14	↓ BP ↓ renal inflammation/damage	Kirabo *et al*.^51^
**mTOR** Rapamycin (mTOR inhibitor)	Dahl salt-sensitive rats fed a 4% NaCl diet for up to 21 days	↓ BP ↓ renal inflammation/damage	Kumar *et al*.^98^
**IMPDH** MMF	SHR Rat	↓ BP ↓ Renal inflammation/damage	Rodríguez-Iturbe *et al*.^99^
MMF	Uni-nephrectomized rats implanted with DOCA-salt pellets + 0.9% NaCl drinking water for up to 21 days.	↓ BP ↓ Renal inflammation/damage	Boesen *et al*.^100^
MMF	Mouse model of SLE	↓ BP ↓ Renal inflammation/damage	Taylor and Ryan^101^
**Purine metabolism** Azathioprine	DOCA-salt in pregnant rats	↓ BP ↓ Proteinuria ↓ Endothelial dysfunction ↓ Systemic inflammation	Tinsley *et al*.^102^

Ab, antibody; ang-II, angiotensin II; BP, blood pressure; CCR, CC motif chemokine receptor; CD, cluster of differentiation; CNI, calcineurin inhibitor; CTLA4-Ig, cytotoxic T-lymphocyte-associated protein 4 immunoglobulin; DOCA, deoxycorticosterone acetate; dTGR, double transgenic rats; IL, interleukin; IFN-γ, interferon γ; ΙMPDH, Inosine-5′-monophosphate dehydrogenase; mTOR: mammalian target of rapamycin; MTX: methotrexate; NF-κb, nuclear factor kappa b; NLRP3, NOD-like receptor family pyrin domain containing 3; PDTC; pyrrolidine dithiocarbamate; SHR, spontaneously hypertensive rat; SLE, systemic lupus erythematosus; TGF-β, transforming growth factor beta; TLR, Toll-like receptor; TNF, tumour necrosis factor.

## 3. Clinical evidence

Epidemiological and observational human data supports a relationship between the immune system and hypertension, including the observation that humans with hypertension are at increased risk of COVID-19 infection-related death.^103^^,^^104^ Inflammatory biomarkers^105–107^ correlate with systolic BP (SBP) in acute stroke, each 10 mmHg BP elevation increasing the odds of an elevated C-reactive protein level by 72%.^108^ Similarly, observational and clinical trial data demonstrate BP increases with each C-reactive protein quartile.^109^^,^^110^ A nested case–control study of 400 normotensive women indicated that the risk of developing hypertension during follow-up increases with higher quartiles of IL-6 and C-reactive protein.^111^ In addition to C-reactive protein and IL-6, TNF-α, IL-1β, IL-18, and CCL2 cytokine levels also appear to be increased in hypertension and may confer risk of developing the disease.^112–118^ These cytokines likely promote cell infiltration, affect renal sodium transport,^75^ and alter vascular function and structure, ultimately leading to sodium and volume retention, increased systemic vascular resistance, and the phenotype of hypertension.

Circulating leucocytes, which are important cellular components of the immune system, show significant perturbations in hypertension. Data from NHANES III demonstrate higher numbers of circulating leucocytes are associated with hypertension.^107^ UK Biobank data similarly indicate that quintile distribution of lymphocyte, monocyte, neutrophil, and eosinophil count is positively associated with BP.^119^ Other studies show that an increased neutrophil to lymphocyte ratio (NLR) predicts development of hypertension.^120–122^ Intermediate and non-classical monocytes are associated with inflammatory states and endothelial dysfunction and are also increased in hypertensive patients.^123–125^ A recent study has shown that signals from the activated endothelium in hypertension induces conversion of classical CD14^++^/CD16^low^ monocytes to CD14^++^CD16^+^ intermediate monocytes. This seems to be mediated by STAT3 activation and associated with increases in IL-6, IL-1β, IL-23, CCL4, and TNF-α.^123^ Monocytes from hypertensive patients also express higher TLR4, and BP control reverses this.^126^

A causal role of lymphocytes in human hypertension is supported by large-scale Mendelian randomization genetic evidence.^119^ T lymphocytes of hypertensive individuals are activated, with increased IL-17A and interferon γ (IFN-γ) production and proportionally higher memory T cells (CD45RO^+^) in adults.^127^ Youn *et al*.^128^ have shown that patients with hypertension have an increased fraction of immunosenescent, proinflammatory, cytotoxic CD8^+^ T cells. Even among hypertensive adolescents, a subset of pro-inflammatory CD4^+^ T cells is associated with SBP and arterial stiffness.^129^ Increased circulating effector memory CD4^+^/CD8^+^ T cells and CD8^+^CD28 null T cells are also present at this early time point in hypertension.^129^^,130^

In summary, clinical studies identify greater proportions of activated pro-inflammatory monocytes and lymphocytes in hypertension. This may promote their infiltration into target organs, leading to perturbations in vascular and renal function, and ultimately modulating BP.

## 4. Genetic and multi-omics evidence

Data from Genome-Wide Association Studies (GWAS) and the transcriptome link hypertension with immune cellular defence and inflammatory responses.^130^^,^^131^ This link is supported by integrative network analysis^132^ and Mendelian randomization approaches,^119^ and is important, considering that heritability of BP is between 33% and 57%.^133–135^

Several GWAS have implicated *SH2B3/LNK* gene in hypertension and myocardial infarction.^130^^,^^132^^,^^136^^,^^137^ SH2B3 encodes a docking protein that seems to be a modulator of T cell activation. Variants of this gene are linked to autoimmune diseases such as multiple sclerosis, coeliac disease, and type 1 diabetes.^137^ Single nucleotide polymorphism (SNP) rs3184504 in SH2B3 is evidential or its' trans-regulatory role in gene expression; regulating 6 out of the 34 BP-related signature genes identified by meta-analysis of GWAS reporting gene expression profiles from 7017 individuals not on anti-hypertensive treatment. All regulated genes are expressed in leucocytes.^138^ Integrative network analysis of BP GWAS with mRNA expression profiles from 3679 participants not on anti-hypertensive agents confirms molecular interactions between key drivers such as SH2B3 and hypertension-related genes.^132^ Mechanistically, T cells from *LNK* knockout mice produce high levels of type I cytokines and these mice exhibit increased sensitivity to angiotensin II (Ang II), leading to hypertension, endothelial and renal dysfunction, increased inflammatory cell infiltrate, and oxidative stress.^139^^,^^140^ Mendelian randomization evidence based on 120 SNPs predictive of leucocyte subpopulations demonstrates a clear, potentially causal, relationship between lymphocyte count and systolic and diastolic BP, while BP itself appears to affect monocyte and neutrophil counts.^119^ Finally, the recent multi-omic kidney analysis uncovered many immunity-related genes (such as IRF5, IRAK1, BP1, TRAF1) whose expression, splicing, and/or methylation ostensibly demonstrate causal relationships with BP.^141^

## 5. Effects of immunomodulatory drugs on BP

Clinically available immunomodulatory drugs employ heterogeneous mechanisms of action, and hence their impact on BP regulatory systems is likely to be diverse. Agents reviewed below are selected to illustrate this breadth.

### 5.1 Selected anti-cytokine therapies

#### 5.1.1 TNF-α inhibitors

**Figure 2:**
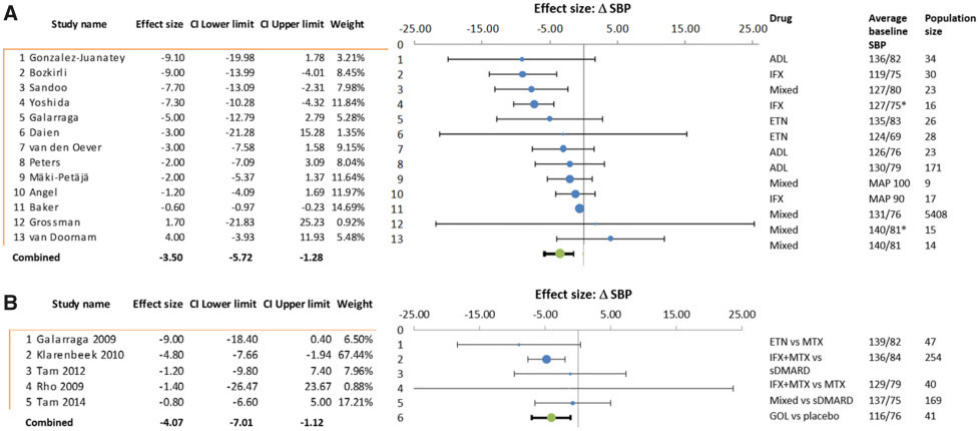
Meta-analysis and Forest Plot using random effect model, of TNF-α inhibitor studies reporting SBP outcomes, with reference to average baseline SBP, population size, and study weighting. Effect size reports average change in SBP in mmHg; * indicates ambulatory BP monitoring and MAP indicates only mean arterial pressure data available. Panel A includes cohort studies reporting average SBP prior and subsequent to drug initiation; panel B includes randomized trials with comparison to placebo or other pharmacotherapy. Overall change in average SBP accompanied by 95% confidence interval. ADL, adalimumab; ETN, etanercept; GOL, golimumab; IFX, infliximab; Mixed, different TNF-α inhibitors within the study; SBP, systolic blood pressure; sDMARD, conventional synthetic disease modifying anti-rheumatic; TNF-α, tumour necrosis factor alpha.

Using a systematized search, we identified 20 studies reporting BP in patients prescribed adalimumab, infliximab, etanercept, golimumab, and six papers with a mix of TNF-α inhibitors used (see *Table 2*). Study populations included those with RA, ankylosing spondylitis, psoriasis, and combined rheumatological diseases. Follow-up was from 2 weeks to 12 months and cohort sizes varied from 9 to 5408. Only 5 of the 20 studies were randomized and/or placebo controlled.^148^^,^^149^^,^^156^^,^^159^ Seventeen of these studies contained data adequate for meta-analysis (see Supplementary material online, methods and *Figure 2*): the combined estimate from 13 studies comparing average BP before and after TNF-α blockade was a 3.5 mmHg reduction in SBP (95% CI: –5.7 to –1.3), *P* = 0.001. Five randomized trials with placebo or other pharmacotherapy comparators produce a combined estimate of 4.1 mmHg SBP lowering (95% CI: –7.0 to –1.1), *P* < 0.001. Only two studies used the gold standard of ambulatory BP monitoring (ABPM), Yoshida *et al*.^146^ demonstrating a SBP reduction of 7.3 mmHg. In contrast Grossman *et al*.^160^ showed that TNF-α blockade caused an increase of 1.7 mmHg. Elevated BP was not an inclusion criterion in any of the studies and hypertension was reported inconsistently. Two of the studies reported only mean arterial pressure,^153^^,^^162^ six studies did not report prevalence or use of anti-hypertensives,^144^^,^^145^^,^^153^^,^^156^^,^^158^^,^^161^ and one specified no anti-hypertensive use.^143^ Hypertension prevalence in the remaining studies ranged between 7% and 53%.^142^^,^^146–152^^,^^157^^,^^159^^,^^160^ In some studies, good BP control was an inclusion criteria.^142^^,^^147^^,^^150^^,^^160^ Finally, individual level data was not available, thus any effect in hypertensive participants may be masked through reporting of average BP across whole study cohorts, though despite this, combined estimates from observational and randomized trials do suggest a BP-lowering effect of anti-TNF-α agents (*Figure 2*). Observational data on incident rates of hypertension offer additional insight. In comparison with non-biologic anti-inflammatory medications, 4822 anti-TNF-α initiators demonstrated no difference in crude or adjusted rates of incident hypertension (HR: 0.95, 95% CI: 0.74–1.2),^163^ results supported by a smaller Korean cohort of 996 patients with RA.^164^ Paradoxically, previous meta-analysis suggested higher rates of incident hypertension as adverse events in TNF-α inhibitor recipients (OR: 1.89, 95% CI: 1.35–2.65).^165^ This disparity indicates need for targeted studies in hypertensive populations.

**Table 2:** Human studies pertaining to TNF-α inhibitor use and reporting data on BP outcomes

References	Population F=female	Design/comparator/follow-up	SBP Baseline mmHg	Δ SBP mmHg *P* value	Notable and confounding features
Gonzalez-Juanatey *et al*.^142^	*n* = 34 (30 F) RA Age 55	Observational: Pre-/post-ADL 52 weeks	136 ± 17.8	–9.1 (–20, 2) *P* = 0.1	9/34 had controlled HTN. Other DMARDs/anti-hypertensives permitted, but no alterations to concomitant medication during study.
Bozkirli *et al*.^143^	*n* = 30 (7 F) Ank Spond Age 34	Pre-/post-IFX 12 weeks	119 ± 9.9	–9 (–14, –4) *P* = 0.001	No anti-hypertensive use. Lower NSAID use at follow up.
Komai *et al*.^144^	*n* = 15 (13F) RA Age 50	Pre-/post-IFX +MTX 6 weeks	127.9 ± 5.6	–8.6 *P* value not reported	Rates of baseline HTN/BP medications unknown. Concomitant MTX/prednisolone doses unknown.
Sandoo *et al*.^145^	*n* = 23 (15 F) RA Age 55	Anti-TNF’s. Control group, *n* = 17 12 weeks	127 ± 15	–7.7 (–20, 5) *P* = 0.007	Rates of baseline HTN/BP medications unknown. Control group (stable on DMARD): no change in BP.
Yoshida *et al*.^146^	*n* = 16 (10 F) RA Age 57	Pre-/post-IFX ± MTX 2 weeks	127.4 ± 21.8	–7.3 (–10, –4) *P* < 0.001	24 h BP. 7/16 HTN; no hypotensive drug administered during study. All on MTX concomitantly, 10/16 on prednisolone.
Galarraga *et al*.^147^	*n* = 26 (22 F) RA Age 57	Pre-/post-ETN: Vs MTX (*n* = 21): 16 weeks	135 ± 16 139 ± 19	–5 (–13, 3) *P* = 0.22 –9 (–18, 0) *P* = 0.06	7/26 controlled HTN. Comparator group on MTX showed no change in BP.
Klarenbeek *et al*.^148^	*n* = 128 (85 F) RA	IFX + MTX Vs sequential monotherapy (*n* = 126) 52 weeks	136 ± 20	–4.8 (–8, –2)* *P* = 0.001	7% of IFX group on anti-hypertensive. *Adjusted for Δ DAS, baseline SBP, age, gender, anti-hypertensive use and Δ BMI. DAS >2.4 associated with higher BP. BP reduction in IFX responders –6.8, non-responders –4.9 mmHg.
Tam *et al*.^149^	*n* = 20 (19 F) RA Age 53	Pre-/post-IFX + MTX: Pre-/post-MTX (*n* = 20): 26 weeks	129 ± 16 130 ± 24	–4.2 ± 13.4 *P* value not reported –3 ± 15 *P* = 0.78*	6/20 HTN. Steroids and other DMARD use permitted. *Between groups comparison of Δ SBP
Daïen *et al*.^150^	N = 28 (28 F) RA Age 57	Pre-/post-ETN 26 weeks	124 ± 15	–3.1 (–22, 16) *P* = 0.55	5/28 controlled HTN. 89% on other sDMARDs. sDMARD group (*n*=20) Δ SBP −1.9 ± 10.9 (NS)
van den Oever *et al*.^151^	*n* = 23 (20 F) RA Age 53	Pre-/post-ADL 26 weeks	126 ± 17	–3.0 (–5, –1) *P* = 0.19	4/28 HTN Osteoarthritis group (*n* = 25), Δ SBP –4 ± 11 (NS)
Peters *et al*.^152^	*n* = 171 (135 F), RA Age 54	Pre-/post-ADL ± DMARD 16 weeks	130 ± 30	–2 (–7, 3) *P* = 0.44	46/171 HTN 133/171 also on MTX and/or other DMARDs
Mäki-Petäjä *et al*.^153^	*n* = 9 RA Age 54	Pre-/post-anti-TNF 12 weeks	MAP 100 ± 9	–2 (–5, 1) *P* = 0.2	Rates of baseline HTN/BP medications unknown. Concomitant drugs: 6/9 steroid, 4/9 DMARD
Rho *et al*.^154^	*n* = 35 RA Age 54	Anti-TNF’s vs. other DMARDs (*n* = 134) Cross-sectional	133.6 ± 21.2	–1.4 (–26, 23) *P* = 0.91	53% of whole cohort (90/169) had HTN, not broken down by drug class.
Angel *et al*.^155^	*n* = 17 (9 F) RA, PsA, Ank Spond Age 54	Pre-/post-anti-IFX 8 weeks	MAP 90 ± 9.1	–1.2 (–4, 2) *P* = 0.37	2/17 HTN (BP had to be well controlled for 6 months). 10/17 on MTX concurrently.
Thaci *et al*.^156^	*n* = 303 Plaque psoriasis	Pre-/post-ETN 52 weeks	126.3	–0.7 *P* value not reported	Rates of baseline HTN/BP medications unknown. Secukinumab and placebo arms, but data only reported for former.
Baker *et al*.^157^	*n* = 5408 RA	Observational pre-/post-anti-TNFs 52 weeks	131 ± 16	–0.6 (–19, 18) *P* value not reported	Data derived from administrative database. 73% HTN and 68% on BP medication at baseline.
Mäki-Petäjä *et al*.^158^	*n* = 17 (11 F) RA Age 58	Pre-/post-ADL or ETN 8 weeks	MAP 104 ± 11	0 *P* = 0.9	9/17 treated HTN
Tam *et al*.^159^	*n* = 20 (92 F) Ank Spond Age 36	GOL: Placebo (*n* = 21): 52 weeks	116 ± 10.4 116 ± 10.1	0.20 ±9.8 0.98 ±8.8 0.8 (–6.6, 5.1)* *P* = 0.79*	2/20 HTN. 7/20 concomitant MTX or sulfasalazine *Between groups comparison of Δ SBP
Grossman *et al*.^160^	*N* = 15 (9 F) RA, PsA, Ank Spond Age 46	Pre-/post-ADL, ETN, or IFX 12 weeks	120.9 ± 11.8	1.7 (–22, 25) *P* = 0.88	ABPM. 2/15 HTN: included if stable for 3 months and BP controlled. 7/15 on other DMARDs.
van Doornum *et al*.^161^	*n* = 14 (8 F) RA Age 55	Pre-/post-anti-TNFs 6 weeks	140 ± 6	4 (–4, 12) *P* = 0.3	Rates of baseline HTN and BP medications not reported. Concurrent DMARD use (MTX 11/14, leflunomide 9/14, HCQ 5/14)

ADL, Adalimumab, ank spod, ankylosing spondylitis; IFX, infliximab; ETN, etanercept; GOL, golimumab; MAP, mean arterial pressure; PsA, psoriatic arthritis; RA, rheumatoid arthritis. Asterix (*) is used to indicate a comment in the final column relating specifically to the asterixed result.

#### 5.1.2 IL-1β inhibition (CANTOS trial)

In a large RCT of patients with prior myocardial infarction and elevated high-sensitivity C-reactive protein, the IL-1β antagonist canakinumab 150 mg demonstrated benefit vs. placebo for a composite end point of myocardial infarction, stroke, or cardiovascular death. Largest effect size was in the quartile demonstrating greatest high-sensitivity C-reactive protein and IL-6 reductions.^10^^,^^166^ Rates of incident hypertension, however, did not differ by high-sensitivity C-reactive protein tertiles; nor did canakinumab demonstrate a reduction in incident hypertension [HR: 0.96 (0.85–1.08), *P* > 0.2]. Ostensibly, this suggests that BP may not be the mechanism by which benefit was exerted; however, baseline prevalence of hypertension was 80%, thus only 20% of participants were ‘at risk’ from incident hypertension.^167^ In the canakinumab arm, only subgroups with baseline BP (SBP ≥130 mm Hg) demonstrated BP lowering, as well as protection from major adverse cardiac events (MACE).^167^ Fatal infections were increased with canakinumab, highlighting the importance of selecting permissible targets.^10^

#### 5.1.3 Anti-IL-17

Considering other cytokine inhibitor approaches, we focused on pharmacotherapies with both animal study evidence and use in clinical practice: secukinumab and tocilizumab. We identified the FIXTURE trial of IL-17 antagonist secukinumab (150 and 300 mg groups) in patients with psoriasis. Despite BP being the primary outcome, this trial reported no change at 1 year, though patients were not hypertensive at baseline.^156^ In contrast, a study of 50 patients with psoriasis commenced on secukinumab demonstrated a 6 mmHg reduction of SBP (130–124 mmHg, *P* = 0.3).^168^ This is particularly important as psoriasis, like RA, appears to be associated with increased prevalence of hypertension and cardiovascular disease.^165^^,^^169^^,^^170^

#### 5.1.4 Anti-IL-6

Three papers were identified reporting BP data with IL-6 antagonist tocilizumab, two used in combination with methotrexate (MTX).^171^^,^^172^ SBP increase was demonstrated by Elmedany *et al.*^171^ (116 ± 16 vs. 129 ± 17 mmHg, *P* = 0.001), the other two papers reporting no change in BP with IL-6 blockade,^172^^,^^173^ though the average baseline BP values were normal range. Overall, the minimally available evidence (detailed in Supplementary material online, *Table*) does not support an association with BP lowering.

### 5.2 Immunosuppressant agents

#### 5.2.1 Mycophenolate mofetil

**Figure 3:**
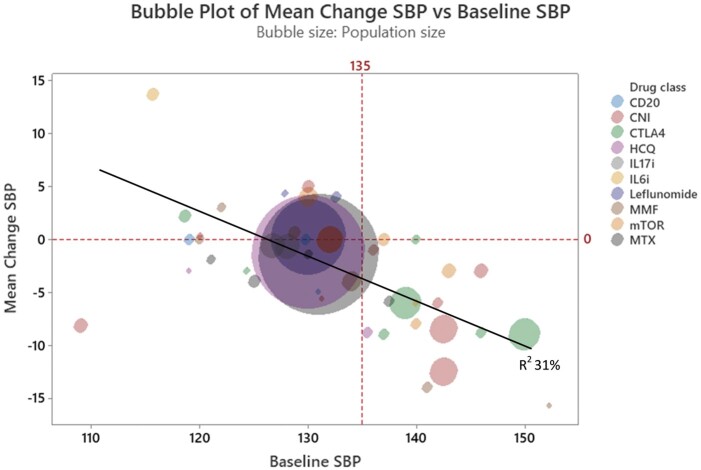
Bubble plot illustrating immunomodulatory agents plotted by baseline SBP (*x*-axis) and average change in SBP (*y*-axis), both in mmHg, with bubble area representing cohort size. *R^2^* = 31% fpr average change in SBP by average baseline SBP. CNI, calcineurin inhibitor; CTLA4-Ig, cytotoxic T-lymphocyte-associated protein 4 immunoglobulin; HCQ, hydroxychloroquine; IL, interleukin; MMF, mycophenolate mofetil; mTOR: mammalian target of rapamycin; MTX: methotrexate; SBP, systolic blood pressure; TNF, tumour necrosis factor.

Mycophenolate mofetil (MMF) inhibits nucleotide synthesis and thus prevents lymphocyte proliferation. In an early study, Herrera *et al.* demonstrated a reduction in average BP from 152/92 to 137/83 mmHg at 12 weeks in eight patients with psoriasis. Notably, BP increased following MMF cessation in this study. The authors also demonstrated a reduction in urinary TNF-α was during MMF therapy.^174^ Other studies reporting BP data are confounded by the presence of nephropathy, with concomitant anti-hypertensive treatment to achieve target BP under 130/80 mmHg, or organ transplantation in which improvement in volume status could obscure any independent impact of MMF on BP. With these caveats in mind, two trials of MMF in IgA nephropathy report BP reduction of –7 to –14 mmHg.^175^^,^^176^ In two other studies of patients with lower enrolment BP, MMF did not reduce SBP beyond treatment with angiotensin-converting enzyme inhibitors (ACEi) alone, or ACEi plus placebo.^177^^,^^178^ Head-to-head trials in transplantation showed that treatment with tacrolimus/MMF lowered SBP by 4 mmHg (*P* = 0.08) and diastolic BP by 3 mmHg (*P* = 0.02) at 6 months compared to transplanted patients receiving tacrolimus/sirolimus. Ninety percent of these subjects had hypertension at baseline.^179^ In a smaller study, no change in BP occurred in 58 liver transplant patients treated with a tacrolimus/MMF (*P* = 0.88, baseline average 129/70 mmHg) whilst a group treated with tacrolimus/steroid showed an 8 mmHg rise in SBP.^180^ Overall, the clinical evidence favours association of MMF with BP reduction in hypertension (see *Figure 3*); however, no data specifically pertaining to hypertensive patients are available.

#### 5.2.2 Methotrexate

MTX is a chemotherapy agent and disease-modifying anti-rheumatic drug (DMARD). Five studies involving between 20 and 8065 participants were identified, reporting average baseline SBP between 121 and 137.5 mmHg. Only one of these employed ABPM. Average SBP lowering ranged from 1.4 to 5.9 mmHg, and diastolic blood pressure (DBP) reduction of up to 4.4 mmHg (see Supplementary material online, *Table* and *Figure 3*).^149^^,^^154^^,^^157^^,^^181^^,^^182^ Conversely, Makavos *et al*.^168^ and CIRT^183^ RCTs in psoriasis and patients with established cardiovascular risk, respectively, did not demonstrate average BP reduction with MTX. Considering the discrepancy, although 90% of the CIRT cohort had hypertension diagnoses, baseline BP values were not reported, precluding assessment of BP effects in individuals with uncontrolled hypertension.

#### 5.2.3 Hydroxychloroquine

Hydroxychloroquine is an antimalarial agent that is used as a DMARD, and experimentally in IgA nephropathy.^154^^,^^157^^,^^184^ This agent has been shown to reduce circulating dendritic cells (DCs) and reduces IFN-α, IL-6, and TNF-α levels.^185^ Three studies of hydroxychloroquine have demonstrated BP lowering. The largest of these involved 7147 patients with RA and showed that hydroxychloroquine lowered BP by 1.2 mmHg systolic/0.6 mmHg diastolic from a baseline of 130/75 mmHg at 6 months.^157^ Two smaller studies report SBP lowering of 3–8.8 mmHg (see Supplementary material online, *Table*).

#### 5.2.4 Leflunomide

Leflunomide is a pyrimidine synthesis inhibitor used in active RA and psoriatic arthritis. In three studies, in which the subjects had an average baseline SBP ranging from 128 to 133 mmHg, a small increase of 1.44–4.3 mmHg systolic and 0.57–4.8 mmHg diastolic in office and ambulatory BP was observed.^154^^,^^157^^,^^186^

#### 5.2.5 Calcineurin inhibitors

**Figure 4:**
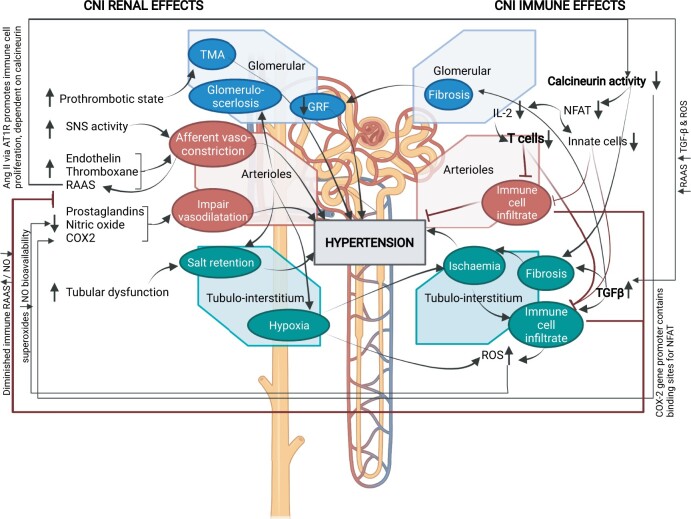
Renal and immune system effects of calcineurin inhibitors influencing blood pressure. COX2, cyclooxygenase-2; GFR, glomerulofiltration rate; IL-2, interleukin-2; NFAT, nuclear factor of activated T cells; NO, nitric oxide; TMA, thrombotic microangiopathy; RAAS, renin–angiotensin–aldosterone system; ROS, reactive oxygen species; SNS, sympathetic nervous system; TGF-β, transforming growth factor beta. Created in BioRender.

Calcineurin inhibitors (CNIs) block the earliest steps of T cell activation, but also have substantial off-target effects, including stimulation of endothelin production, increases in sympathetic outflow, renal vasoconstriction, salt retention, and hypertension (*Figure 4*).^187^^,^^188^ Eight studies with BP data following 12–36 months of CNI treatment are reviewed (see Supplementary material online). In four of these, the baseline BP was in the hypertensive range.^189–192^ Six reported lowering of BP (range –1 to –13 mmHg),^189–194^ while two demonstrated a rise in BP of 5–11 mmHg.^168^^,^^195^ Further detail is available in the Supplementary material online and reviewed elsewhere,^188^ but in summary, limitations of trial design, and CNI off target effects make interpretation of BP effects of CNIs difficult (*Figure 4*).

#### 5.2.6 Mammalian target of rapamycin inhibitors

Mammalian target of rapamycin (mTOR) inhibitors such as sirolimus and everolimus regulate cellular metabolism, growth, and proliferation, offering alternative immunosuppression following transplantation. Of six studies we found reporting BP values, the three reporting an average baseline SBP >140 mmHg all suggested a reduction in BP of between 3 and 8 mmHg,^189^^,^^190^^,^^192^ though only one achieved statistical significance.^189^ ABPM was only measured in the SCHEDULE trial of heart transplant patients treated with everolimus. An 8 mmHg fall in SBP (*P* = 0.05), and no change in DBP occurred from 2 weeks post-transplant to 12 months follow-up. This was dominated by reduction in nocturnal SBP in both the everolimus and cyclosporine arms.^189^ BP lowering was not observed when the average baseline BP for the study was in the normotensive range.^195^ mTOR inhibitors in comparison with other agents reported SBP outcomes that were neutral or elevated (0 or +4 mmHg change).^179^^,^^195^^,^^196^ These studies are complicated by the concomitant use of multiple other drugs (see Supplementary material online).

#### 5.2.7 Cytotoxic T-lymphocyte-associated protein 4-Ig

Abatacept is composed of the Fc region of the immunoglobulin IgG1 fused to the extracellular domain of cytotoxic T-lymphocyte-associated protein 4 (CTLA-4). This agent targets T cell co-stimulation and is commonly used in transplant and rheumatologic diseases. In five studies of RA patients reporting BP outcomes with abatacept, specific values were not available for two and none of the others reported a statistically significant effect on BP.^171^^,^^173^^,^^197–199^ Seven studies reporting BP outcomes using Belatacept, an alternative CTLA-Ig, were identified. All of these were in transplant recipients and were compared to patients receiving CNIs. Two of these studies involved cross over from CNI to Belatacept and showed a SBP reduction of 5.4 and 8.8 mmHg (*P* = 0.38 and 0.03, respectively).^200^^,^^201^ A case–control study reported a 9 mmHg lower SBP in subjects treated with this agent (*P* = 0.68).^202^ Three RCTs showed a reduction in SBP between –2.4 and –9 mmHg,^203–205^ but only one of these reached statistical significance^204^ (see Supplementary material online, *Table*). One RCT reported no difference in mean SBP.^206^ In only two studies did the subjects have an average baseline SBP in the hypertensive range,^199^^,^^201^ and no studies employed ABPM. The apparent BP benefit with belatacept but not abatacept likely reflects population differences (transplant vs. RA, respectively), potential physiological changes post-transplantation, and the cross-over effect from CNI, which as noted above, has off-target effects that can raise BP.

#### 5.2.8 Rituximab

Rituximab is a monoclonal antibody against CD20, resulting in B cell apoptosis and depletion. It is used in lymphoid and blood malignancies and diverse autoimmune diseases*.* Trials reporting BP that are not confounded by polypharmacy were sparse. We identified four such studies, and none involved patient groups with uncontrolled hypertension—the average participant baseline SBP being 131/83 mmHg or lower.^173^^,^^207–209^ An early reduction in BP is common, but data reporting longer-term trends were discordant. No BP effect was seen in RA^173^^,^^207^^,^^208^; but a BP reduction was observed in membranous nephropathy at 4 weeks, though not sustained to 20 weeks.^209^

### 5.3 Determinants of the BP effects of immunosuppressants

**Figure 5:**
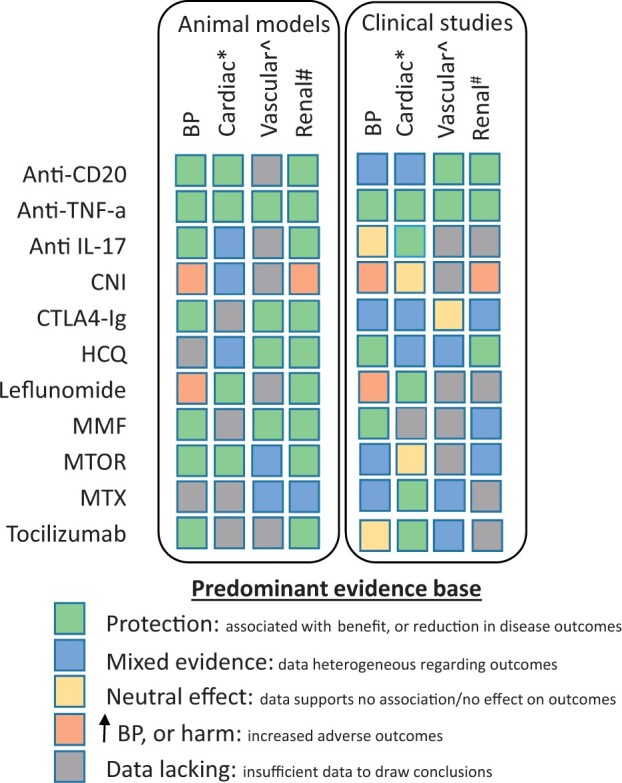
Immunomodulatory drugs and the level of animal and clinical evidence available regarding blood pressure and organ system outcomes. Summarized according to the aggregated weight of the available evidence. BP, blood pressure; CD, cluster of differentiation; CNI, calcineurin inhibitor; CTLA4-Ig, cytotoxic T-lymphocyte-associated protein 4 immunoglobulin; HCQ, hydroxychloroquine; IL, interleukin; MMF, mycophenolate mofetil; mTOR: mammalian target of rapamycin; MTX: methotrexate; TNF, tumour necrosis factor. *Cardiovascular outcomes. ^Includes arterial stiffness, endothelial function, and cerebrovascular outcomes. ^#^Includes chronic kidney disease, end-stage kidney disease, fibrosis, and inflammation.

In summary, trials in rheumatic, autoimmune, and transplant patients indicate a possible BP-lowering effect of selected anti-inflammatory therapies targeting diverse pathways previously identified by pre-clinical studies. The evidence appears to be most consistent in relation to anti-TNF-α agents, while other therapies such as hydroxychloroquine, MMF, and mTORs all suggest BP-lowering effect (*Figures 3 and 5*). Data are however conflicting, and hypertension was rarely a pre-specified outcome measure. Trials often involved normotensive populations in which BP lowering is difficult to observe. A combined analysis of studies discussed in this paper shows that cohorts with higher average baseline SBP appear to achieve greater BP-lowering effect (*Figure 3*), an association also reported for anti-hypertensive drugs.^210–212^

### 5.4 Non-pharmacological interventions

Several *non*-*pharmacological treatment* approaches have shown beneficial effects in reducing inflammation and therefore improving patient outcomes in the context of hypertension.

#### 5.4.1 Periodontitis targeting and BP

Animal studies suggest that periodontal *Porphyromonas gingivalis* infection increases IFN-γ and TNF-α production through modulation of Th1 responses, leading to BP elevation, endothelial dysfunction, and vascular inflammation.^213^ This link is supported by Mendelian randomization,^214^ observational data, and meta-analysis.^215–218^ Data from well-controlled trials demonstrate that intense treatment of periodontitis can improve endothelial function,^219^ lower inflammatory markers, and BP as measured by ABPM, with a reduction in SBP of 5 mmHg (*P* < 0.01).^220^ A meta-analysis of eight studies involving intensive periodontal treatment showed an average decrease of SBP of −4.3 mm Hg (95% CI: –9.1 to –0.5) and DBP –3.16 mm Hg (95% CI: –6.5 to –0.2), though none of these achieved statistical significance. As in the case of pharmacological interventions, BP reductions were not observed in normotensive individuals.^221^

#### 5.4.2 Energy metabolism, microbiome, and salt

Physical activity has an established role in BP regulation, with 3 months of exercise lowering SBP by approximately 5 mmHg and DBP by 3 mmHg.^222^ Physical activity also has demonstrable immune effects.^223^ Exercise can both increase circulating numbers of T cells^224^ and improve response to influenza vaccination.^225^

For dietary interventions, most research has focused on CVD risk reduction, though BP lowering has also been demonstrated in both normotensive and hypertensive cohorts,^226^^,^^227^ at least in part immune-mediated via effects of diet on the microbiome.^228^ Metagenome-wide association evidence of gut dysbiosis in hypertension includes restricted sample diversity, higher lipopolysaccharide synthesis, membrane transport, and steroid degradation; suggesting low-grade inflammatory stimuli may be the mechanism.^229^ Evidence is accumulating that plant-based dietary protein may promote bacterial species associated with anti-inflammatory effects, while meat consumption is linked to CVD and inflammatory bowel disease.^230^

Dietary salt is another dominant driver of hypertension, primarily through activation of renin–angiotensin–aldosterone system^231^; at higher concentration, salt also favours pro-inflammatory monocyte^232^ and T cell phenotypes with increased tissue infiltration^233^ and microvascular dysfunction.^234^ Highly controlled experimental reduction in salt intake lowers pro-inflammatory IL-6 and IL-23, and increases IL-10 levels,^235^ though this effect was not detected in a larger observational study.^236^

#### 5.4.3 Neuronal manipulations

The central nervous system regulates vascular and kidney function through sympathetic innervation but is also a potent modulator of immune responses. Animal and human studies demonstrate the role of neuroimmune axis in the pathogenesis of hypertension,^237^^,^^238^ with murine renal denervation (RDN) inducing a reduction in BP,^239–242^ and reduction in renal inflammation, T cell activation, and pro-inflammatory cytokine production.^240^^,^^243^ However, SIMPLICITY, SPYRAL, and RADIANCE human clinical trials demonstrate inconsistent results, mostly favouring sustained BP reduction.^244^^,^^245^ Some but not all^246^ of these were sham-controlled RDN designs.^247–249^ The effect of RDN on immune activation in humans is less clear. One trial demonstrated reductions in TNF-α and IL-1β, and up-regulation of IL-10 one day after RDN; however, this did not persist to day 3,^250^ and was not corroborated elsewhere.^251^^,^^252^

An alternative approach to sympathetic denervation is augmentation of parasympathetic activity through vagus nerve stimulation (VNS). This approach has proven effective in hypertensive rodent models.^253–255^ VNS limits hypertension-induced endothelial dysfunction^256^ and reduces levels of systemic cytokines and mRNA expression in target organs,^257^ with both afferent and efferent VNS protecting mice from kidney injury.^258^ Anti-inflammatory effects of VNS are abolished in immune-deficient and β2 adrenergic receptor-deficient mice.^259^ When stimulated by Ang II or bioelectronic signals, a splenic neuroimmune cascade is triggered via α-adrenergic receptors. In response, CD8^+^ effector T cells with a role in hypertension^25^ egress from the spleen.^260^ VNS has not yet been tested in human hypertension, but has been used in epilepsy and in RA, demonstrating lowering of circulating TNF-α, IL-1-β, and IL-6 levels and improvement in disease activity.^261^

### 5.5 Hypertension-mediated organ damage

Hypertension-mediated organ damage (HMOD) correlates with BP values in hypertension^262^^,^^263^; however, genetics, lifestyle, and co-morbid conditions may also contribute to end-organ damage independently of BP levels. Similarly, the target organ benefit of immunomodulation might be partially independent of BP effects. The strength of evidence regarding the effects of immunomodulatory therapy on HMOD in experimental and clinical settings is summarized in *Figure 5*. Registry data of active RA, with 30% hypertension prevalence, suggested no difference in myocardial infarction rates in response to TNF-α inhibitors vs. DMARDs. However, 60% fewer events did occur in the TNF-α inhibitor responder subgroup vs. non-responders.^264^ The observational QUEST-RA study included subjects with hypertension prevalence of 32% and was adjusted for traditional risk factors. This study reported a reduction in cardiovascular risk in response to numerous immunomodulatory drugs, including biologic agents (HR: 0.42; 95% CI: 0.21–0.81), MTX (HR: 0.85; 95% CI: 0.81–0.89), sulfasalazine (HR: 0.92; 95% CI: 0.87–0.98), and leflunomide (HR: 0.59; 95% CI: 0.43–0.79); *P* < 0.05.^265^ Baseline BP values were not reported in either paper. Nurmohamed *et al.* reviewed 90 studies reporting cardiovascular risk outcomes in rheumatological conditions treated with abatacept, TNF-α inhibitors, rituximab, secukinumab, tocilizumab, and tofacitinib. They report a neutral effect on BP, on surrogate markers of cardiovascular risk, and on MACE, though authors emphasise the variation in quantity and quality of evidence.^266^

Observational data based on 13 000 matched pairs from Medicare and MarketScan patients with RA and newly treated with abatacept or a TNF-α inhibitor found benefit of abatacept in MACE restricted to the subgroup with diabetes [HR: 0.74 (95% CI: 0.57–0.96)].^267^ Potential confounding arose from higher rates of hypertension in the diabetic subgroups, again supporting potential benefit of TNF-α inhibitors in hypertension.^167^ Finally, a meta-analysis of 14 studies in patients with RA, adjusted for hypertension, concluded that DMARDs were associated with an increased risk of MACE relative to TNF-α inhibitor therapy [OR: 1.58 (95% CI: 1.16–2.15); I2 = 16%], effect maintained in presence or absence of MTX.^268^

Colchicine is hypothesized to inhibit microtubular polymerization, assembly of the NLRP3 inflammasome, and IL-1β and IL-18 production. In acute coronary syndrome, colchicine abrogates local increases in IL-1β, IL-18, and IL-6 levels,^269^ and its addition to aspirin and statin reduces high-sensitivity C-reactive protein.^270^ Colchicine 0.5 mg daily has been demonstrated to reduce MACE by 67% compared to placebo in LoDoCo RCT of 532 patients with stable coronary artery disease,^271^ though the similarly sized COPS trial found no benefit in cardiovascular outcomes.^272^ The larger COLCOT trial of 4745 participants recruited within 30 days of acute coronary syndrome reported composite cardiovascular end-point occurrences in 5.5% of the colchicine group vs. 7.1% of the placebo group (HR: 0.77; 95% CI: 0.61–0.96; *P* = 0.02).^9^ Half of these patients had hypertension. Similarly, LoDoCo2 randomized 5522 chronic coronary disease patients to low-dose colchicine, with composite end-point events in 6.8% of the colchicine group vs. 9.6% of placebo group (HR: 0.69; 95% CI: 0.57–0.83; *P* < 0.001).^11^

Overall, we would conclude that there is evidence of improvement in MACE for TNF-α inhibitors, MTX, tocilizumab, secukinumab, leflunomide and colchicine, though heterogeneity of study designs and outcomes limits the strength of this statement, and we have not explored the relationship between reduction in inflammation and MACE suggested by CANTOS and TNF-α inhibitor responders in the registry data above. HMOD outcomes beyond MACE are surmised in *Figure 5* for common immunomodulatory drugs.

## 6. Conclusions

While experimental, genetic, and clinical evidence supports the role of inflammation and immune system involvement in hypertension and associated vascular, renal, and cardiac pathology, immunomodulatory approaches are not currently considered therapeutic options in BP lowering and cardiovascular disease reduction. Indeed, clinical evidence reviewed in this paper shown a highly heterogeneous effect of immune targeting on BP and cardiovascular events across a wide range of patients mainly with various underlying immune-mediated diseases. Going forward, there are several important considerations. As is the case with traditional anti-hypertensive medications, the BP-lowering effects of anti-inflammatory agents appear to be limited to those with uncontrolled hypertension. This is not surprising as numerous compensatory mechanisms make lowering beyond normal BP difficult. It is also important to consider that the effects may be limited to patients with active pro-hypertensive inflammatory mechanisms. The lesson from CIRT, TNF-α inhibitor responders vs. non-responders, CANTOS, and the body of the evidence presented is that there must be *active* inflammation. Hence, cardiovascular risk reduction with immune modulation is mediated not through BP alone, but via broader mechanisms of oxidative stress, endothelial function, vascular remodelling, and endocrine regulation, that are the ‘common denominators’ of a dysfunctional relationship. Secondly, we must target the optimal checkpoint in the inflammation–hypertension relationship to optimize benefit without adverse effect, and so far, this has remained elusive at a population level. Finally, it is important to consider that virtually all of the preclinical studies investigating the anti-hypertensive effect of immune interventions on hypertension have involved treatment of animals at the onset on hypertension, often concomitantly with the onset of the disease. In contrast, these agents are usually given to humans with long-standing hypertension. It is possible, and even likely that once hypertension has been established, there are chronic changes in renal and vascular function and structure that render such treatment less effective. In this regard, treatment of younger individuals with early onset hypertension might yield different results than those observed in the studies summarized here.

## Supplementary material

Supplementary material is available at *Cardiovascular Research* online.

**Conflict of interest:** none to declare. 

## Funding

This work was supported by the European Research Council (Project Identifier: 726318); the British Heart Foundation grants PG/19/84/34771, PG/21/10541, and RE/13/5/30177; the Marie Sklodowska Curie CIG Award 3631773; the Wellcome Trust grant 204820/Z/16/Z; and the University of Glasgow, Scottish Funding Council and the Global Challenges Research Fund.

## Data availability

Data derived from sources in the public domain. Reference details are provided in full.

## Supplementary Material

cvab330_Supplementary_Data

## Notes

This article is part of the Spotlight Issue on Cardiovascular Immunology.

Tomasz Guzik and Pasquale Maffia have positions within CVR; the manuscript was handled by a consulting editor.
