# Supplementary material for: Therapeutic targeting of inflammation in hypertension: from novel mechanisms to translational perspective
Source: Cardiovasc Res. 2021 Oct 26;117(13):2589–609. doi: 10.1093/cvr/cvab330 (PMC9825256; doi:10.1093/cvr/cvab330)
Supplement: cvab330_Supplementary_Data [file cvab330_supplementary_data.pdf]

Therapeutic targeting of inflammation in hypertension: from novel mechanisms to translational perspective

Eleanor C. Murray [1] , Ryszard Nosalski [1,2], Neil MacRitchie [3] , Maciej Tomaszewski [4,5], Pasquale Maffia [1,3,6], David G. Harrison [7] , and Tomasz J. Guzik [1,2]*

[1] Institute of Cardiovascular and Medical Sciences, College of Medical, Veterinary and Life Sciences, University of Glasgow, G12 8TA Glasgow, UK; [2] Department of Internal Medicine, Collegium Medicum, Jagiellonian University, 31-008 Krako´w, Poland; [3] Centre for Immunobiology, Institute of Infection, Immunity and Inflammation, College of Medical, Veterinary and Life Sciences, University of Glasgow, G12 8TA Glasgow, UK; [4] Division of Cardiovascular Sciences, Faculty of Medicine, Biology and Health, University of Manchester, M13 9PL Manchester, UK; [5] Manchester Heart Centre and Manchester Academic Health Science Centre, Manchester University NHS Foundation Trust, M13 9WL Manchester, UK; [6] Department of Pharmacy, University of Naples Federico II, 80131 Naples, Italy; and [7] Division of Clinical Pharmacology, Department of Medicine, Vanderbildt University Medical Centre, Nashville, 37232 TN, USA

Online Supplementary data

Methods

To evaluate evidence regarding the role of inflammation in hypertension we conducted a review of data pertaining to 1) BP outcomes with immunomodulatory medications, selected for inclusion on their ability to illustrate a broad range of pharmacological classes, 2) effects of antihypertensive pharmacological agents on immune and inflammatory parameters, and 3) non-pharmacological approaches targeting inflammation in hypertension. The systematized approach was adopted, as data was largely not adequate to complete meta-analysis according to PRISMA requirements. The population comprised any disease group requiring immunomodulatory medication (the intervention), with comparisons where possible of placebo groups, and normotensive versus hypertensive. Data extracted included design of study including use of randomisation or placebo-control; cohort size, therapeutic agent and dose; duration of follow up with 12 month data chosen if numerous time points available, baseline blood pressure values, change in blood pressure (and any statistical analysis of), and other cardiovascular outcome measures. Ethical approval was not required due to the review nature of the study.

With regard to the human data only, Embase and Pubmed Search Strategies included search terms: blood pressure, hypertens*, inflammatory disease, transplant*, effect, impact, action, tacrolimus, ciclosporin, abatacept, belatacept, rituximab, mycophenolate, basiliximab, infliximab, etanercept, tocilizumab, vascular stiffness, PWV. Furthermore, we also searched ClinicaTrials.Gov for “hypertension” to capture additional studies actively recruiting, or as of yet unpublished. 479 registered trials were screened; 31 in detail, one contributing to the publication. Papers published subsequent to the date of the literature search (03/05/2019) were included if deemed critically relevant to the topic and otherwise met the criteria.

Studies were excluded if duplicates, if based on animal models, participant number was 5 or less, they were review articles (though systematic reviews are referred to if offering additional perspective), were not directly relevant e.g. referred to pulmonary hypertension. Studies of potential value but without reported blood pressure values were contacted to request blood pressure data, though not always successfully.

Adequate number of studies and data were available for meta-analysis of TNF-α inhibitors alone; protocol for assessing inclusion eligibility was as follows:

1) Full-length publication in peer-reviewed journal, or abstract presented at international meeting.

2) Administration of TNF- α inhibitor for a minimum of 6 weeks, for any disease indication.

3) Cross-over, placebo-controlled, and head-to-head comparison studies included.

4) Other immunomodulatory medications not an exclusion if adequately controlled for.

5) Data retrieved included proportion with hypertension or on anti-hypertensive medications; baseline and follow up blood pressure (systolic and diastolic), change in BP and confidence interval as published, or calculated.

6) Minimum number of participants 5; case reports excluded.

On the basis of this protocol, 880 abstracts were reviewed pertaining to TNF-α inhibitors and BP outcomes; 862 excluded on the above grounds; 2 added from search of citations and subsequent publications, and final number included in qualitative synthesis of the paper totaled 20.

Supplementary Table. Blood pressure outcomes of therapeutic agents targeting the immune system.

Drug class Reference	Population	Design (Observational unless specified) / follow-up / comparator	SBP Baseline	SBP Treated	Δ SBP (95% CI, or SD)	P value	Notable and confounding features
HCQ<br>Rho 2009 [1]	N=42<br>RA<br>Age 54	Current use (cross-sectional)<br>Vs other DMARDs (n=134)	136 ± 20	127 ± 21	-8.8	0.01	53% of whole cohort (90/169) had HTN, not broken down by drug class.<br>Beta (adjusted for known confounders) -4.59 (-9.99–0.82), P = 0.1
HCQ<br>Baker 2018 [2]	N= 7147 (15% F)<br>RA<br>Age 63	Observational (database interrogation)<br>26 wks<br>Pre-/post-HCQ	130 ± 17	Not reported	-1.2	Not reported	77% HTN<br>Based on proportion with optimal BP, MTX RR 1.09 P<0.0001; Leflunomide RR 0.97 (NS); HCQ RR 1.07 P<0.0001; TNFi RR 1.05 P<0.05.<br>Multivariable Model evaluating Δ SBP: MTX as reference; Leflunomide β 1.82 (1.2 to 2.5) P<0.001; TNFi β 0.9 (0.3 to 1.5) p=0.003; HCQ β -0.31 (-0.9 to 0.3) NS.
HCQ<br>Gao 2017 [3]	N=14 (9 F)<br>IgAN<br>Age 39	FU 52 wks<br>Pre/post-HCQ<br>Pre/post Losartan comparator	119 ±12<br>116 ± 14	116 ± 9<br>119 ± 10	-3	NS<br>NS	All on losartan (standard care)<br>Neither pre-/post-HCQ nor between-group differences statistically significant.
RTX<br>Provan 2015 [4]	N=24 (17 F)<br>RA<br>Age 57	Observational;12 wks<br>Pre/post RTX<br>Pre/post ABT comparator	128 ± 16<br>109 ± 11		-1.3 ± 10.1<br>4.0 ± 9.6	0.53<br>0.4	RTX vs ABT Δ SBP 0.85 (beta -8.8 (-14.6,-3)

RTX<br>Novikova 2016 [5]	N=55 (55 F)<br>RA<br>Age 50	26 wks<br>Pre/post-RTX	119 ± 2.8*	119 ± 2.4*	0	NS	Concurrent DMARDS in 47/55, steroids in 44/55, NSAIDs in 54/55<br>* BP in RTX responder subgroup, n= 41 (non-responder group: 112 ± 2.8, to 125 ± 2.4)
RTX<br>Mathieu 2012 [6]	N=33 (29F)<br>RA<br>Age 61	52 wks<br>Pre/post-RTX	130 ± 21		'No change'		26/33 concurrent DMARDs<br>13/33 concurrent anti-hypertensive
RTX<br>Remuzzi 2002 [7]	N=8 (4 F)<br>Membranous Nephropathy<br>Age 52	4 wks<br>Pre/post-RTX	131 ± 2	136 ± 4	-5	NS	BP likely reflects disease treatment, with SBP back to baseline by week 20 (130 ± 5 mmHg)
CNI<br>Andreassen 2019 [8]	N=43 (12 F)<br>Cardiac Tx<br>Age 51	Randomised<br>52 wks<br>Pre/post-CIC:<br>EVR comparator (n=40):	136 ± 16*<br><br>140 ± 14*	135 ± 10<br><br>132 ± 12	-1 (-17,15)<br><br>-8 (-23, 7)	NS<br><br>0.05	*Baseline BP recorded at 2 weeks may reduce confounding from early physiological changes. Used ABPM.<br>EVR arm also on CIC until week 7 to 11. Concurrent MMF and steroids.<br>Δ SBP 8 mmHg more in the EVR arm vs CIC (95%CI 0, 15), P = 0.05.<br>Antihypertensive drug use: CIC 80% to 90%; EVR 78% to 69%, P= 0.14
CNI<br>Fijter 2017 [9]	N=356 (104 F; 125 CIC and 231 TAC)<br>Kidney Tx<br>Age 47	Randomised; 2yrs<br>vs EVR (n=359)<br>comparator	132	132	0	NS	HTN as cause of ESRD equal both groups.<br>HTN as adverse event during FU equal both groups.<br>Concomitant mycophenolic acid and steroids.
CNI<br>Chamienia 2014 [10]	Kidney Tx<br>N=14 (5 F); age 41<br>N=15 (7 F); age 46	Randomised; 2yrs<br>Pre/post high-TAC<br><br>Pre/post low-TAC	131 ± 15<br><br>120 ± 12	126 ± 11<br><br>120 ± 14	-5.6<br><br>0.3	NS<br><br>NS	HTN as cause of ESRD equal both groups.<br>Difference in TAC levels between groups lost by 24 months.<br>BP reported at multiple time points, with variability by FU period and no consistent difference between groups.
CNI<br>Larson 2006 [11]	N=84 (40 F)<br>Kidney Tx<br>Age 48	Randomised; 52 wks<br>Pre/post TAC<br>vs SRL (n=81)	130 ± 20<br>137 ± 15	135 ± 22<br>135 ± 22	5<br>-2	Not reported<br>0.56	Antihypertensive drugs could be commenced, but proportion of patients on drugs fell over the study period
CNI<br>Murbraech 2015 [12]	N=27 (9 F)<br>Kidney Tx<br>Age 58	Randomised; 3yrs<br>Pre/post CIC<br>Pre/post EVR comparator	142 ± 15<br>140 ± 14	136 ± 13<br>134 ± 12	-6<br>-6	0.08<br>0.14	Mixed model for difference between groups from baseline to 3 yrs FU P=0.96<br>No difference in antihypertensive use (P= 0.97) between groups of time-points.
CNI<br>Claes 2012 [13]	N=1645 (33-38% F)<br>Kidney Tx<br>Age 46	Randomised; 52 wks<br>Std CIC (n= 390)<br>Low-CIC (n= 399)<br>Low-TAC (n= 401)	144<br>143<br>143	133<br>134<br>130	-11<br>-10<br>-13	Low-CIC vs low-TAC -4 mmHg, P<0.05*	Concomitant MMF and corticosteroids. Daclizumab induction to all patients except Std-CIC group.<br>Antihypertensive drug use: 77%, no between group difference, P=0.61<br>* After adjustment for multiple comparisons

	± metabolic syndrome	Low-SRL (n= 399)	144	131	-13		
CNI<br>Rostaing 2012 [14]	N=339 (27 F)<br>Kidney Tx<br>HTN subgroup<br>n=92; age 57	Multi-centre, single arm 24 wk cross-over from CIC to TAC<br>HTN subgroup:	109		-5 (-6, -4)<br>-8.2 (-11, -6)	Not reported<br>Not reported	92/339 HTN; over half taking lipid lowering and/or antihypertensive medication at baseline.<br>Concurrent MMF and steroid as standard.<br>No change baseline to 24wks in number of antihypertensive drugs.
CNI<br>Van Dijk 2018 [15]	N=89 (56 F)<br>Kidney Tx<br>Age 50	Randomised. 2 yrs<br>Pre/post CIC<br>Pre/post EVR comparator* (n=96)<br>Pre/post MPS* (n=39)	146 ± 20<br>143 ± 19<br>143 ± 18	143 ± 22<br>140 ± 17<br>146 ± 20	-3*<br>-3*<br>3*	NS<br>NS<br>NS	ESRD due to HTN in 16.5%. Basiliximab induction; CIC, MPS, prednisolone until randomization at 6 months.<br>Mean number of antihypertensives 1.95 (±1.28) to 2.08 (±1.07) P<0.005<br>*Between groups P=0.37
CNI<br>Makavos 2020 [16]	N=50 (18 F, age 53)<br>Psoriasis	Randomised to SEC (N=50), CIC, or MTX (N=50)<br>52 wks FU<br>Pre/post CIC	125 ± 15	136 ± 10	11	0.03*	Similar rates of baseline hypertension across groups (28-32%)<br>No between group statistical comparisons made<br>* Bonferroni-adjusted P value
CNI<br>Mourer 2013 [17]	N=59 (18 F)<br>Kidney Tx<br>Age 54	Randomised; 3 yrs<br>CNI withdrawal:<br>MMF withdrawal comparator (n=50):	128 ± 12<br>128 ± 14	121 ± 9<br>129 ± 10	-6.6<br>-0.2	Not reported<br>NS	ABPM. >60% on BP medications.<br>Difference between the groups at FU: P=0.004.<br>Decline in BP in CNI withdrawal (slope daytime SBP, -1.6 mm Hg/y, P=0.018) not seen in MMF withdrawal.
CNI<br>Cicinnati 2007 [18]	N=50 (15 F)<br>Liver Tx<br>Age 54	Randomised; 52 wks CNI reduction:<br>vs continuation (n=25):	133 ± 18<br>131 ± 17	125 ± 13<br>131 ± 15	-8.5<br>-0.9	0.001<br>NS	>1 yr since transplant. Used ABPM<br>MMF up-titrated, then CNI tapered to trough levels 2–4 ng ⁄mL (TAC) or 25–50 ng ⁄mL (CIC).
CNI<br>Schrama 2000 [19]	N= 15 (9 F)<br>Kidney Tx<br>Age 47	Open, prospective, pre-/post-CNI withdrawal<br>8 wks	152 ± 13	145 ± 13	-7	<0.01	CIC tapered (stopped by 32 wks); MMF and 7.5mg prednisolone continued.<br>ABPM.
CTLA4<br>Ursini 2015 [20]	N=15 (7 F)<br>RA<br>Age 53	Observational; 24 wks Pre-/post-ABT	124 ± 13	121 ± 10	-3	0.45	Concomitant DMARDs (all on MTX, 4/15 on HCQ) but no prior biologics.<br>5/15 on ACEi/ARB.
CTLA4<br>Mathieu 2013 [21]	N=21 (17 F)<br>RA<br>Age 65	Observational, 26 wks Pre-/ post-ABT	140 ± 22		'No change'		17/21 on DMARDs ± NSAIDs in TNFi non-responders
CTLA4	N=5 (5 F)	12 wks			4 ± 9.6	0.4	RTX vs ABT Δ SBP 0.85 (beta -8.8 (-14.6,-3)

Provan 2015 [4]	RA<br>Age 54	Pre- / post- ABT:<br>Pre/post RTX comparator:	109 ± 11<br>128 ± 16		-1.3 ± 10.1	0.53	
CTLA4<br>Elmedany<br>2019[22]	N=60 (60 F)<br>RA<br>Age 48	Randomised; 24 wks<br>Pre/post-ABT*<br>Pre/post-TCZ*	119 ± 15<br>116 ± 16	121 ± 14<br>129 ± 17	2.2<br>13.7	0.36<br>0.001	Concurrent MTX ± steroids/NSAIDs. Prior TNFi use similar across groups.<br>*Between group difference SBP at FU 8.5mmHg P = 0.002
CTLA4<br>Iasella 2018 [23]	N=11 (4F, age 54)<br>Lung Tx, CNI<br>'failure'	Conversion CNI to<br>BELAT; MAP<br>Median 19 wks FU	98	92	-5.4	0.38	Induction therapy: almetuzumab or basiliximab; maintenance: TAC, MMF, and prednisolone, with TAC to BELAT switch as intervention<br>Baseline HTN rates unknown
CTLA4<br>Malvezzi 2019<br>[24]	N=35 (8F, age 56)<br>Kidney Tx	Conversion CIC to BELAT<br>52 wks	146 ± 19	138 ± 16	-8.8	0.3	Median time Tx to conversion to BELAT was 3.3 years.<br>Maintainance MMF ± steroids<br>23 reduced antihypertensives, 12 had doses increases.
CTLA4<br>Vincenti 2010<br>[25]	N=527 (31/35% F,<br>age 44/43 by<br>respective group)<br>Kidney Tx	52 wks<br>Pre-/post more intensive<br>BELAT (n=173):<br>Less intensive (n=181):<br>vs CIC n=173	139<br>139	133 ± 16<br>131± 17	-6<br>-9	0.027*	* both BELAT groups vs CIC group<br>35% CIC arm vs 26-29% BELAT groups on ≥3 antihypertensives, p=0.02
CTLA4<br>Seibert 2014 [26]	N=46 (13 F, age 54)<br>Kidney Tx	Cross sectional data; min 20<br>months post-Tx<br>BELAT vs CIC:	137 (IQR<br>121-147)	128 (IQR<br>116-152)	-9	0.68	Baseline HTN 100% CIC group, vs 87% BELAT group
CTLA4<br>Durrbach 2010<br>[27]	N=543 (35/26% F,<br>age 57/56 by<br>respective BELAT<br>group)<br>Kidney Tx	52 wks<br>BELAT (n=359) vs CIC<br>(n=184):	150	141	-9	Not<br>reported	52% CIC arm vs 39–43% BELAT groups on ≥3 antihypertensives
MTX<br>Daien 2013 [28]	N=20 (20 F)<br>RA<br>Age 51	Observational cohort. 26<br>wks FU<br>Pre/post-DMARD<br>Pre/post-ETN n=28	121 ± 13<br>124 ± 15		-1.9 ± 10.9<br>-3.1 ± 18.8	NS	Normotensive<br>* DMARD: MTX, sulfasalazine, or LFN
MTX Mangoni<br>2017 [29]	N=56 (39 F)<br>RA<br>Age 61	Observational; 32 wk<br>a) Pre- / post-MTX<br>b) MTX vs no-MTX*	125 ± 3	121 ± 3	-4<br>-7.7 (-13.2,<br>-2.3)*	0.006	ABPM<br>* Adjusted for age, gender, BMI, and disease activity score

MTX<br>Rho 2009 [1]	N= 31<br>RA<br>Age 54	Cross-sectional comparison between DMARD classes:-<br>No LFN vs LFN (n=31)<br>No MTX vs MTX (n=49)	133 ± 20<br>138 ± 18	137 ± 20<br>132 ± 21	4<br>-5.9	0.28<br>0.09	Beta (adjusted for known confounders) 5.7 (-0.32–11.73), P = 0.07<br>Beta (adjusted for known confounders) -1.35 (-6.67–3.97) P= 0.62.
MTX<br>Rozman 2002 [30]	N=17<br>RA	Observational; 26 wk<br>Pre/post LFN	128 ± 19	132 ± 21	4.3	0.003	ABPM<br>± low dose steroid/NSAID
MTX<br>Baker 2018 [2]	N= 8065 MTX (13% F, age 63)<br>N= 3035 LFN (12%, age 64)<br>RA	Observational (database); 26 wks.<br>Pre/post-MTX<br>Pre/post-LFN	131 ± 17<br>130 ± 17		-1.4<br>0.2	Not reported	MTX 74% HTN; LFN 75% HTN<br>Based on proportion with optimal BP, MTX RR 1.09 P<0.0001; Lefunomide RR 0.97 (NS); HCQ RR 1.07 P<0.0001; TNFi RR 1.05 P<0.05.<br>Multivariable Model evaluating Δ SBP: MTX as reference; Lefunomide β1.82 (1.2, 2.5) P<0.001; TNFi β0.9 (0.3, 1.5) p=0.003; HCQ β -0.31 (-0.9, 0.3) NS.
MTX<br>Gyldenløve 2015 [31]	N= 32 (16F, age 46)<br>Psoriasis	Observational; 8-10 wks<br>Pre/post-MTX	127<br>(95-160)	125<br>(95-165)	-2	0.944	16% hypertension at baseline
MTX Makavos 2020[16]	N=50 (20 F, age 53)<br>Psoriasis	Randomised to SEC (n=50), CIC (n=50), or MTX<br>52 wks FU<br>Pre/post MTX:	128 ± 10	130 ± 10	2	0.7*	Similar rates of baseline hypertension across groups (28-32%)<br>No between group statistical comparisons made<br>* Bonferroni-adjusted P value
MTX<br>Tam 2012[32]	N=20 (15 F)<br>RA<br>Age 53	Randomised; 26 wks<br>Pre/post MTX:<br>Pre/post MTX+IFX:	130 ± 24<br>129 ± 16	127 ± 15<br>125	-3 ± 15<br>–4.2 ± 13	0.79*	*Comparison between the changes from baseline between the 2 groups
mTOR<br>Fijter 2017[9]	N=359 (114 F)<br>Kidney Tx<br>Age 46	Randomised; 2yrs<br>vs CNI (n=356) comparator	132	132	0	NS	HTN as cause of ESRD equal both groups.<br>HTN as adverse event during FU equal both groups.<br>Concomitant mycophenolic acid and steroids.
mTOR<br>Andreassen 2019[8]	N=40 (9 F)<br>Cardiac Tx<br>Age 51	Randomised; 52 wks<br>Pre/post-EVR:<br>Pre/post-CNI (n=43):	140 ± 14*<br>136 ± 16*	132 ± 12<br>135 ± 10	-8 (-23, 7)<br>-1 (-17,15)	0.05<br>NS	*Baseline ABPM recorded at 2 weeks.<br>EVR arm also on CIC until wk 7 to 11. All on MMF and steroids.<br>EVR arm Δ SBP 8 mmHg than CIC (95%CI 0, 15), P = 0.05.<br>Antihypertensive drugs: CIC 80% to 90%; EVR 78% to 69%, NS
mTOR<br>Gonwa 2003[33]	N=185 (62F)<br>Kidney Tx<br>Age 45	Randomised; multicentre; 26 wks<br>MMF vs SRL	130 ± 19	134 ± 18	4	0.08	Baseline HTN SRL 28.6%, MMF 30.7%.<br>Both groups with concomitant TAC

mTOR<br>Larson 2006[11]	N=81 (36 F)<br>Kidney Tx<br>Age 50	Randomised, 52 wks<br>Pre/post-SRL:<br>vs TAC (n=84):	137 ± 15<br>130 ± 20	135 ± 22<br>135 ± 22	-2<br>5	0.56<br>Not<br>reported	Antihypertensive drugs could be commenced, but proportion of patients on drugs fell over the study period
mTOR<br>Murbraech<br>2015[12]	N=17 (10 F)<br>Kidney Tx<br>Age 61	Randomised; 3yrs<br>Pre-/post CIC<br>Pre-/post EVR	142 ± 15<br>140 ± 14	136 ± 13<br>134 ± 12	-6<br>-6	0.08<br>0.14	Mixed model: no difference between groups (P=0.96)<br>No difference in antihypertensive use (P= 0.97) between groups.
mTOR<br>Van Dijk<br>2018[15]	N=96 (49 F)<br>Kidney Tx<br>Age 51	Randomised; 2yrs<br>Pre/post EVR:<br>Pre/post CNI (n=89):<br>Pre/post MPS (n=39):	146 ± 20<br>143 ± 19<br>143 ± 18	143 ± 22<br>140 ± 17<br>146 ± 20	-3*<br>-3*<br>3*	NS<br>NS<br>NS	ESRD due to HTN in 16.5%.<br>Basiliximab induction; CIC, MPS and steroid until randomized at 6 mo.<br>*Between group difference P=0.37
mTOR<br>Gonwa 2003[33]	N=176 (53 F)<br>Kidney Tx<br>Age 48	Randomised; 26 wks<br>MMF vs SRL	134 ± 18	130 ± 19	-4	0.08	Both groups with TAC.<br>HTN as cause of ESRD in 31% MMF arm vs 29% SRL.
MMF<br>Herrera 2006[34]	N=8 (5 F)<br>Psoriasis and RA + HTN<br>Age 50-65	Observational. 12 wks FU (1 month prior to, during, and after cessation of MMF)	152 ± 6.6	137 ± 5	-15.7	<0.001	4/8 on MTX, discontinued 2 wks previously.<br>4/8 on anti-hypertensives at baseline.<br>BP reverted after MMF stopped.
MMF<br>Mourer 2013[17]	N=60 (21 F)<br>Kidney Tx<br>Age 52	Randomised. 3yrs<br>CNI withdrawal (n=59):<br>MMF withdrawal:	128 ± 12<br>128 ± 14	121 ± 9<br>129 ± 10	-6.6<br>-0.2	Not<br>reported<br>NS	ABPM. >60% on BP medications.<br>Difference between the groups at FU: P=0.004.<br>Decline in BP in CNI withdrawal (slope daytime SBP, -1.6 mm Hg/y, P=0.018) not seen in MMF withdrawal.
MMF<br>Maes 2004[35]	N=21 (5 F)<br>IgAN<br>Age 39	Randomised. 3yrs<br>Pre/post MMF<br>Pre/post placebo	122 ± 4<br>134 ± 8	125 ± 3<br>124 ± 8	3<br>-10	*	6/21 on anti-hypertensives already. All started on ACEi as standard. Enalapril dose twice as high in the MMF arm vs placebo (19 vs 11mg) P <0.05.<br>*Linear mixed model treatment effect 0.12; P= 0.72.
MMF<br>Tang 2010[36]	N=20 (14 F)<br>IgAN<br>Age 42	Randomised. 6yrs<br>Pre/post-MMF:<br>Comparator ACEi alone (n=20):	120<br>122	121<br>121	1<br>-1	NS<br>NS	All on ACEi/ARB as standard.<br>1.4 anti-hypertensives MMF arm, vs 1.7 control arm.
MMF<br>Liu 2014 [37]	N=42 (18F)<br>IgAN<br>Age 40	Randomised; 1.5 yrs<br>Pre/post MMF<br>Pre/post control	141 ± 15<br>134 ± 18	127 ± 12*<br>125 ± 10*	-14<br>-9	Not<br>reported	All on ACEi/ARB as standard.<br>Control group: CIC and prednisolone, n=42<br>* Between group difference P=0.336
MMF<br>Frisch 2005 [38]	N=17<br>IgAN	Randomised; 2yrs<br>Pre/post MMF:	136 ± 19	129 ± 12	-7	Not<br>reported	All on ACEi/ARB ± other antihypertensives to target <130, and higher baseline BP in MMF arm - reduction likely just reflects study protocol to achieve target BP

	Age 39	Pre/post placebo: (n=15)	131 ± 11	128 ± 6	-3		
MMF<br>Pascual 2006 [39]	N=246<br>Kidney Tx	Randomised, multicentre.<br>3yrs FU<br>MMF w/d vs control arm<br>n=237	136	140	3.6	0.002	Single office BP reading.<br>Antihypertensive use at FU: control arm (CNI/MMF/steroid) 66.2%; MMF withdrawal arm 74.4%; P 0.008). Mean number antihypertensives: 1.8 vs 2.0 respectively.
MMF<br>Cuervas-Mons 2015[40]	N=58 (12 F)<br>Liver Tx<br>Age 56	52 wks<br>Pre/post-MMF:<br>vs pre-/post steroid: (n=59)	129 ± 25<br>124 ± 17	129 ± 22<br>132 ± 18	0.6<br>7.9	0.88<br><0.01	Both arms with concomitant TAC.<br>Baseline HTN 17% vs 31%. New onset HTN 30.6% (steroid) vs 42.5% (MMF).<br>Antihypertensive use not reported.
Interleukin antagonist<br>Thaci 2016 [41]	Psoriatic arthritis<br>N=312 300mg<br>N=315 150mg	Randomised. 52 wks<br>Pre/post SEC:<br>vs ETN n=303:	126.7<br>128.1	126.1<br>127.4	-0.6<br>-0.7	NS	Demographics and baseline characteristics comparable across groups
Interleukin antagonist<br>Makavos 2020[16]	N=50 (20 F, age 51)<br>Psoriasis	Randomised to SEC or CIC (N=50), or MTX (N=50)<br>52 wks FU<br>Pre/post SEC	130 ± 10	124 ± 8	-6	0.3*	Similar rates of baseline hypertension across groups (28-32%)<br>No between group statistical comparisons made<br>* Bonferroni-adjusted P value
Interleukin antagonist<br>CANTOS<br>Rothman 2020[42]	N=9549 (25-27% F*)<br>MI with hsCRP >2mg/L<br>Age 59-64*	Canakinumab<br>Randomised vs placebo<br>52 wks	130	Not reported		>0.2	* Average for different quartiles. 80% HTN at baseline<br>Canakinumab did not reduce SBP at 3-, 6-, or 12-months, P>0.2<br>Did not reduce incident HTN (HR 0.96 [0.85–1.08], P>0.2), but did reduce MACE.<br>Rates of incident HTN were 23.4, 26.6, and 28.1/100-person years for lowest to highest hsCRP (P>0.2).
Interleukin antagonist<br>Provan 2015 [4]	N=7 (6 F)<br>RA<br>Age 52	12 wks<br>Pre/post TCZ<br>Pre/post- ABT (n=5)	133 ± 22<br>109 ± 11	Not reported	-11.5 ± 18.6<br>4 ± 9.6	0.15<br>0.4	RTX vs ABT Δ SBP 0.85 (beta -8.8 (-14.6,-3)
Interleukin antagonist<br>Elmedany 2019[22]	N=58 (58 F)<br>RA<br>Age 51	Randomised; 24 wks<br>Pre/post-ABT*<br>Pre/post-TCZ*	119 ± 15<br>116 ± 16	121 ± 14<br>129 ± 17	2.2<br>13.7	0.36<br>0.001	Concurrent MTX ± steroids/NSAIDs. Prior TNFi use similar across groups.<br>*Between group difference SBP at FU 8.5mmHg P = 0.002

Studies of immunomodulatory medications in humans reporting SBP outcomes; grouped by mechanism of action. Age: reported average age; FU: follow up; wks: weeks; HCQ: hydoxychlooquine; RTX: rituximab; TCZ: tocilizumab; Tx: Transplant; mTOR: mammalian target of rapamycin; EVR: everolimus; SRL: sirolimus; RR: relative risk; ARR: absolute risk reduction; MTX: methotrexate; LFN: leflunomide; MPS: mycophenolate sodium; BELAT: Belatacept; SEC: Secukinumab. Design – 'Pre/post': average SBP before and following introduction of the drug; 'drug comparator': BP values before and after introduction of alternate drug are provided for comparison; 'Vs drug' = difference between groups reported. SBP: mean ± SD.

1. Rho YH, Oeser A, Chung CP, Milne GL, Stein CM. Drugs used in the treatment of rheumatoid arthritis: Relationship between current use and cardiovascular risk factors. *Arch Drug Inf* 2009;**2**:34–40.
2. Baker JF, Sauer B, Teng CC, George M, Cannon GW, Ibrahim S, Cannella A, England BR, Michaud K, Caplan L, Davis LA, O'Dell J, Mikuls TR. Initiation of Disease-Modifying Therapies in Rheumatoid Arthritis Is Associated with Changes in Blood Pressure. *J Clin Rheumatol* 2018;**24**:203–209.
3. Gao R, Wu W, Wen Y, Li X. Hydroxychloroquine alleviates persistent proteinuria in IgA nephropathy. *Int Urol Nephrol* 2017;
4. Provan SA, Berg IJ, Hammer HB, Mathiessen A, Kvien TK, Semb AG. The impact of newer biological disease modifying anti-rheumatic drugs on cardiovascular risk factors: A 12-month longitudinal study in rheumatoid arthritis patients treated with rituximab, abatacept and tociliziumab. *PLoS One* 2015;
5. Novikova DS, Popkova T V., Lukina G V., Luchikhina EL, Karateev DE, Volkov A V., Novikov AA, Aleksandrova EN, Nasonov EL. The effects of rituximab on lipids, arterial stiffness, and carotid intima-Media thickness in rheumatoid arthritis. *J Korean Med Sci* 2016;
6. Mathieu S, Pereira B, Dubost JJ, Lusson JR, Soubrier M. No significant change in arterial stiffness in RA after 6 months and 1 year of rituximab treatment. *Rheumatol (United Kingdom)* 2012;**51**:1107–1111.
7. Remuzzi G, Chiurchiu C, Abbate M, Brusegan V, Bontempelli M, Ruggenenti P. Rituximab for idiopathic membranous nephropathy. *Lancet* 2002;
8. Andreassen AK, Broch K, Eiskjær H, Karason K, Gude E, Mølbak D, Stueflotten W, Gullestad L. Blood Pressure in De Novo Heart Transplant Recipients Treated With Everolimus Compared With a Cyclosporine-based Regimen. *Transplantation* Ovid Technologies (Wolters Kluwer Health); 2019;**103**:781–788.
9. Fijter JW de, Holdaas H, Øyen O, Sanders JS, Sundar S, Bemelman FJ, Sommerer C, Pascual J, Avihingsanon Y, Pongskul C, Oppenheimer F, Toselli L, Russ G, Wang Z, Lopez P, Kochuparampil J, Cruzado JM, Giet M van der, Gaite LE, Lopez VF, Maldonado R, Massari P, Novoa P, Palti G, Chadban S, Kanellis J, Masterson R, Oberbauer R, Saemann M, Kuypers D, et al. Early Conversion From Calcineurin Inhibitor- to Everolimus-Based Therapy Following Kidney Transplantation: Results of the Randomized ELEVATE Trial. *Am J Transplant* 2017;
10. Chamienia A, Biedunkiewicz B, Król E, Dębska-Ślizień A, Rutkowski B. One-year observation of kidney allograft recipients converted from cyclosporine microemulsion to tacrolimus. *Transplantation Proceedings* 2006.
11. Larson TS, Dean PG, Stegall MD, Griffin MD, Textor SC, Schwab TR, Gloor JM, Cosio FG, Lund WJ, Kremers WK, Nyberg SL, Ishitani MB, Prieto M, Velosa JA. Complete avoidance of calcineurin inhibitors in renal transplantation: A randomized trial comparing sirolimus and tacrolimus. *Am J Transplant* 2006;
12. Murbraech K, Massey R, Undset LH, Midtvedt K, Holdaas H, Aakhus S. Cardiac response to early conversion from calcineurin inhibitor to everolimus in renal transplant recipients - a three-yr serial echocardiographic substudy of the randomized controlled CENTRAL trial. *Clin Transplant* 2015;
13. Claes K, Meier-Kriesche HU, Schold JD, Vanrenterghem Y, Halloran PF, Ekberg H. Effect of different immunosuppressive regimens on the evolution of distinct metabolic parameters: Evidence from the Symphony study. *Nephrol Dial Transplant* 2012;
14. Rostaing L, Sánchez-Fructuoso A, Franco A, Glyda M, Kuypers DR, Jaray J. Conversion to tacrolimus once-daily from ciclosporin in stable kidney transplant recipients: a multicenter study. *Transpl Int* John Wiley & Sons, Ltd; 2012;**25**:391–400.
15. Dijk M van, Roon AM van, Said MY, Bemelman FJ, Homan van der Heide JJ, Fijter HW de, Vries APJ de, Bakker SJL, Sanders JSF. Long-term cardiovascular outcome of renal transplant recipients after

early conversion to everolimus compared to calcineurin inhibition: results from the randomized controlled MECANO trial. *Transpl Int* 2018;

16. Makavos G, Ikonomidis I, Andreadou I, Varoudi M, Kapniari I, Loukeri E, Theodoropoulos K, Pavlidis G, Triantafyllidi H, Thymis J, Parissis J, Tsoumani M, Rafouli-Stergiou P, Katsimbri P, Papadavid E. Effects of Interleukin 17A Inhibition on Myocardial Deformation and Vascular Function in Psoriasis. *Can J Cardiol* Elsevier Inc.; 2020;**36**:100–111.

17. Mourer JSJ, Koning EEJP De, Zwet EWE van, Mallat MJKM, Rabelink TTJ, Fijter JW De, JW de F, Fijter JW De. Impact of late calcineurin inhibitor withdrawal on ambulatory blood pressure and carotid intima media thickness in renal transplant recipients. *Transplantation* 2013;**96**:49–57.

18. Cicinnati VR, Yu Z, Klein CG, Sotiropoulos GC, Saner F, Malago M, Frilling A, Gerken G, Brolesh CE, Beckebaum S. Clinical trial: switch to combined mycophenolate mofetil and minimal dose calcineurin inhibitor in stable liver transplant patients - assessment of renal and allograft function, cardiovascular risk factors and immune monitoring. *Aliment Pharmacol Ther* John Wiley & Sons, Ltd; 2007;**26**:1195–1208.

19. Schrama Y, Joles J, Tol A van, Boer P, Koomans H, Hene RJ. Conversion to Mycophenolate Mofetil in conjuction with stepwise withdrawal of cyclosporin in stable renal transplant recipients. *Transplantation* 2000;**69**:376–383.

20. Ursini F, Russo E, Hribal ML, Mauro D, Savarino F, Bruno C, Tripolino C, Rubino M, Naty S, Grembiale RD. Abatacept Improves Whole-Body Insulin Sensitivity in Rheumatoid Arthritis. *Med (United States)* 2015;

21. Mathieu S, Couderc M, Glace B, Pereira B, Tournadre A, Dubost JJ, Soubrier M. Effects of 6 months of abatacept treatment on aortic stiffness in patients with rheumatoid arthritis. *Biol Targets Ther* 2013;

22. Elmedany SH, Mohamed AE, Galil SMA. Efficacy and safety profile of intravenous tocilizumab versus intravenous abatacept in treating female Saudi Arabian patients with active moderate-to-severe rheumatoid arthritis. *Clin Rheumatol* 2019;

23. Iasella CJ, Winstead RJ, Moore CA, Johnson BA, Feinberg AT, Morrell MR, Hayanga JWA, Lendermon EA, Zeevi A, McDyer JF, Ensor CR. Maintenance Belatacept-Based Immunosuppression in Lung Transplantation Recipients Who Failed Calcineurin Inhibitors. *Transplantation* 2018;

24. Malvezzi P, Fischman C, Rigault G, Jacob MC, Raskovalova T, Jouve T, Janbon B, Rostaing L, Cravedi P. Switching renal transplant recipients to belatacept therapy: results of a real-life gradual conversion protocol. *Transpl Immunol* 2019;

25. Vincenti F, Charpentier B, Vanrenterghem Y, Rostaing L, Bresnahan B, Darji P, Massari P, Mondragon-Ramirez GA, Agarwal M, Russo G Di, Lin C-S, Garg P, Larsen CP. A Phase III Study of Belatacept-based Immunosuppression Regimens versus Cyclosporine in Renal Transplant Recipients (BENEFIT Study). *Am J Transplant* Blackwell Publishing Ltd; 2010;**10**:535–546.

26. Seibert FS, Steltzer J, Melilli E, Grannas G, Pagonas N, Bauer F, Zidek W, Grinyó J, Westhoff TH. Differential impact of belatacept and cyclosporine A on central aortic blood pressure and arterial stiffness after renal transplantation. *Clin Transplant* 2014;

27. Durrbach A, Pestana JM, Pearson T, Vincenti F, Garcia VD, Campistol J, Carmen Rial M Del, Florman S, Block A, Russo G Di, Xing J, Garg P, Grinyó J. A phase III study of belatacept versus cyclosporine in kidney transplants from extended criteria donors (BENEFIT-EXT Study). *Am J Transplant* Blackwell Publishing Ltd; 2010;**10**:547–557.

28. Daïen CI, Fesler P, Cailar G Du, Daïen V, Mura T, Dupuy AM, Cristol JP, Ribstein J, Combe B, Morel J. Etanercept normalises left ventricular mass in patients with rheumatoid arthritis. *Ann Rheum Dis* BMJ Publishing Group Ltd; 2013;**72**:881–887.

29. Mangoni AA, Baghdadi LR, Shanahan EM, Wiese MD, Tommasi S, Elliot D, Woodman RJ. Methotrexate, blood pressure and markers of arterial function in patients with rheumatoid arthritis: a repeated

cross-sectional study. *Ther Adv Musculoskelet Dis* 2017;

30. Rozman B, Praprotnik S, Logar D, Tomšič M, Hojnik M, Kos-Golja M, Accetto R, Dolenc P. Leflunomide and hypertension. Ann. Rheum. Dis. BMJ Publishing Group Ltd; 2002. p. 567–569.

31. Gyldenløve M, Jensen P, Løvendorf MB, Zachariae C, Hansen PR, Skov L. Short-term treatment with methotrexate does not affect microvascular endothelial function in patients with psoriasis. *J Eur Acad Dermatology Venereol* 2015;

32. Tam LS, Shang Q, Li EK, Wang S, Li RJ, Lee KL, Leung YY, Ying KY, Yim CW, Kun EW, Leung MH, Li M, Li TK, Zhu TY, Chui RK, Tseung L, Yu SL, Kuan WP, Yu CM. Infliximab is associated with improvement in arterial stiffness in patients with early rheumatoid arthritis - A randomized trial. *J Rheumatol* 2012;

33. Gonwa T, Mendez R, Yang HC, Weinstein S, Jensik S, Steinberg S. Randomized trial of tacrolimus in combination with sirolimus or mycophenolate mofetil in kidney transplantation: Results at 6 months. *Transplantation* 2003.

34. Herrera J, Ferrebuz A, MacGregor EG, Rodriguez-Iturbe B. Mycophenolate Mofetil Treatment Improves Hypertension in Patients with Psoriasis and Rheumatoid Arthritis. *JASN* American Society of Nephrology (ASN); 2006;**17**:S218–S225.

35. Maes BD, Oyen R, Claes K, Evenepoel P, Kuypers D, Vanwalleghem J, Damme B Van, Ch Vanrenterghem YF, Vanrenterghem YFC. Mycophenolate mofetil in IgA nephropathy: Results of a 3-year prospective placebo-controlled randomized study. *Kidney International* 2004. p. 1842–1849.

36. Tang SCW, Tang AWC, Wong SSH, Leung JCK, Ho YW, Lai KN. Long-term study of mycophenolate mofetil treatment in IgA nephropathy. *Kidney Int* 2010;**77**:543–549.

37. Liu X, Dewei D, Sun S, Xu G, Liu H, He L, Zhang P. Treatment of severe IgA nephropathy: Mycophenolate mofetil/prednisone compared to cyclophosphamide/prednisone. *Int J Clin Pharmacol Ther* 2014;**52**:95–102.

38. Frisch G, Lin J, Rosenstock J, Markowitz G, Agati VD', Radhakrishnan J, Preddie D, Crew J, Valeri A, Appel G. Mycophenolate mofetil (MMF) vs placebo in patients with moderately advanced IgA nephropathy: a double-blind randomized controlled trial. *Nephrol Dial Transpl* 2005;**20**:2139–2145.

39. Pascual J, Hooff JP Van, Salmela K, Lang P, Rigotti P, Budde K. Three-year observational follow-up of a multicenter, randomized trial on tacrolimus-based therapy with withdrawal of steroids or mycophenolate mofetil after renal transplant. *Transplantation* 2006;

40. Cuervas-Mons V, Herrero JI, Gomez MA, González-Pinto I, Serrano T, la Mata M de, Fabregat J, Gastaca M, Bilbao I, Varo E, Sánchez-Antolín G, Rodrigo J, Espinosa MD. Impact of tacrolimus and mycophenolate mofetil regimen vs. a conventional therapy with steroids on cardiovascular risk in liver transplant patients. *Clin Transplant* 2015;**29**:667–677.

41. Thaci D, Girolomoni G, Philipp S, Qureshi A, You R, Fox T. Secukinumab treatment does not induce blood pressure change in subjects with moderate to severe plaque psoriasis: Results from the FIXTURE study. *J Am Acad Dermatol* Elsevier; 2016;**74**:AB273.

42. Rothman AMK, MacFadyen J, Thuren T, Webb A, Harrison DG, Guzik TJ, Libby P, Glynn RJ, Ridker PM. Effects of Interleukin-1β Inhibition on Blood Pressure, Incident Hypertension, and Residual Inflammatory Risk: A Secondary Analysis of CANTOS. *Hypertension* Lippincott Williams and Wilkins; 2020;**75**:477–482.
